# Gamma frequency light flicker regulates amyloid precursor protein trafficking for reducing β‐amyloid load in Alzheimer's disease model

**DOI:** 10.1111/acel.13573

**Published:** 2022-02-23

**Authors:** Qi Shen, Xiaolei Wu, Zhan Zhang, Di Zhang, Sihua Yang, Da Xing

**Affiliations:** ^1^ MOE Key Laboratory of Laser Life Science & Institute of Laser Life Science South China Normal University Guangzhou China; ^2^ College of Biophotonics South China Normal University Guangzhou China

**Keywords:** Alzheimer's disease, amyloid precursor protein trafficking, GABA_A_ receptor α1, gamma frequency light flicker, KCC2, β‐amyloid

## Abstract

Inducing gamma oscillations with non‐invasive light flicker has been reported to impact Alzheimer's disease‐related pathology. However, it is unclear which signaling pathways are involved in reducing amyloid load. Here, we found that gamma frequency light flicker increased anchoring of amyloid precursor protein (APP) to the plasma membrane for non‐amyloidogenic processing, and then physically interacted with KCC2, a neuron‐specific K^+^‐Cl^−^ cotransporter, suggesting that it is essential to maintain surface GABA_A_ receptor α1 levels and reduce β‐amyloid (Aβ) production. Stimulation with such light flicker limited KCC2 internalization and subsequent degradation via both tyrosine phosphorylation and ubiquitination, leading to an increase in surface‐KCC2 levels. Specifically, PKC‐dependent phosphorylation of APP on a serine residue was induced by gamma frequency light flicker, which was responsible for maintaining plasma membrane levels of full‐length APP, leading to its reduced trafficking to endosomes and inhibiting the β‐secretase cleavage pathway. The activated PKC from the gamma frequency light flicker subsequently phosphorylated serine of KCC2 and stabilized it onto the cell surface, which contributed to the upregulation of surface GABA_A_ receptor α1 levels. Together, these data indicate that enhancement of APP trafficking to the plasma membrane via light flicker plays a critical modulatory role in reduction of Aβ load in Alzheimer's disease.

## Graphical abstract

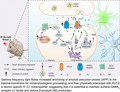

## 1 INTRODUCTION

Gamma oscillations occur between 20 and 50 Hz and represent a form of neural‐network activity in many areas of the brain, which are closely related to learning, memory consolidation, and recall (Herrmann & Mecklinger, 2001; Tallon‐Baudry & Bertrand, 1999; Tiitinen et al., 1993). Altered neuronal gamma oscillations have been observed in multiple brain regions in several neurological and psychiatric disorders, including reduced gamma power in multiple Alzheimer's disease (AD) mouse models and human AD patients (Gillespie et al., 2016; Ribary et al., 1992; Stam et al., 2002; Verret et al., 2012). Notably, changing neural activity has been shown to impact AD pathology, such as β‐amyloid (Aβ) and tau accumulation (Bero et al., 2011; Verret et al., 2012; Wu et al., 2016).

Extracellular Aβ deposition is a pathologic hallmark of AD and is a central tenet of the decades‐old “amyloid‐cascade hypothesis,” which posits that neuronal dysfunction, synaptic loss, neurofibrillary degeneration, and the full manifestation of AD neuropathology are initiated by aberrant Aβ deposition (Das et al., 2015). Aβ is generated by sequential proteolytic processing of amyloid precursor protein (APP) by the enzymes β‐ and γ‐secretases, with β‐site APP‐cleaving enzyme‐1 (BACE‐1) cleavage being the rate‐limiting step in this pathway (O'Brien & Wong, 2011; Z. Zhang et al., 2020). It has been shown that APP is a ubiquitous transmembrane protein that is cleaved during its subcellular trafficking when co‐compartmentalizing at specific locations with active APP secretases (Small & Sam, 2006). Indeed, Aβ production mainly occurs at the Golgi apparatus and endosomes (Choy et al., 2012). However, full‐length APP may be alternatively cleaved by α‐secretase which occurs between the Golgi apparatus and the plasma membrane (PM) (Yan et al., 2001). Recently, a non‐invasive 40 Hz light‐flickering regime has been demonstrated to reduce Aβ_1‐40_ and Aβ_1‐42_ levels, as well as the levels of cleavage intermediates of APP, implying that these may be achieved by altering APP trafficking (Iaccarino et al., 2016; Martorell et al., 2019). However, the underlying flicker‐induced responses to AD‐related molecular and cellular pathology remain unclear. Thus, it appears that non‐invasive 40 Hz light flicker may induce a neuroprotective response that alters general endosomal processing of APP.

Synaptic GABA_A_ receptors (GABA_A_Rs) normally tightly regulate synaptic signaling by reducing the ability of the receiving neuron to respond, but this inhibition is disrupted in AD; hence, there is a potential mechanism for APP to regulate GABAergic signaling and synaptic inhibition (Braat & Kooy, 2015). The neuronal‐specific K^+^‐Cl^−^ cotransporter, KCC2, maintains low intracellular Cl^−^ concentrations in the adult brain, which is essential for maintaining postsynaptic inhibition mediated by GABA_A_Rs (Kaila et al., 2014; Rivera et al., 1999). It has been shown that full‐length APP maintains normal GABAergic inhibition via a direct protein–protein interaction with KCC2 (Chen et al., 2017). Although exogenous GABAergic antagonism completely abrogated the effects of 40 Hz light flicker on Aβ levels which presumably results from a blockade of gamma oscillations, it is largely unknown as to which signaling pathways regulate GABA_A_R‐mediated signaling and interactions between APP and KCC2 during such treatment with 40 Hz light flicker.

In the current study, we aimed to detect whether and how gamma frequency light flicker affects APP processing in AD to reduce Aβ burden, and regulates GABA_A_R‐mediated signaling through KCC2. We show that flicker at gamma frequency increased transport of APP from the Golgi apparatus to the PM, which then physically interacted with KCC2 to restore surface GABA_A_R α1 levels and reduce Aβ production. Specifically, phosphorylation of APP on a serine residue induced by protein kinase C (PKC) activation under the treatment of gamma frequency light flicker led to the maintenance of plasma membrane levels of full‐length APP, and also decreased APP trafficking to endosomes to ultimately inhibiting BACE1 cleavage. Moreover, on the basis of PKC‐induced serine phosphorylation of KCC2, the tyrosine phosphorylation and degradation of KCC2 were further limited by a direct interaction with full‐length APP anchored within the PM. Taken together, these data indicate that enhancement of APP trafficking to the PM via light flicker plays a key regulatory role in maintaining surface GABA_A_R α1 levels and reducing Aβ load.

## 2 RESULTS

### 2.1 Gamma frequency light flicker reduces amyloid load

**FIGURE 1:**
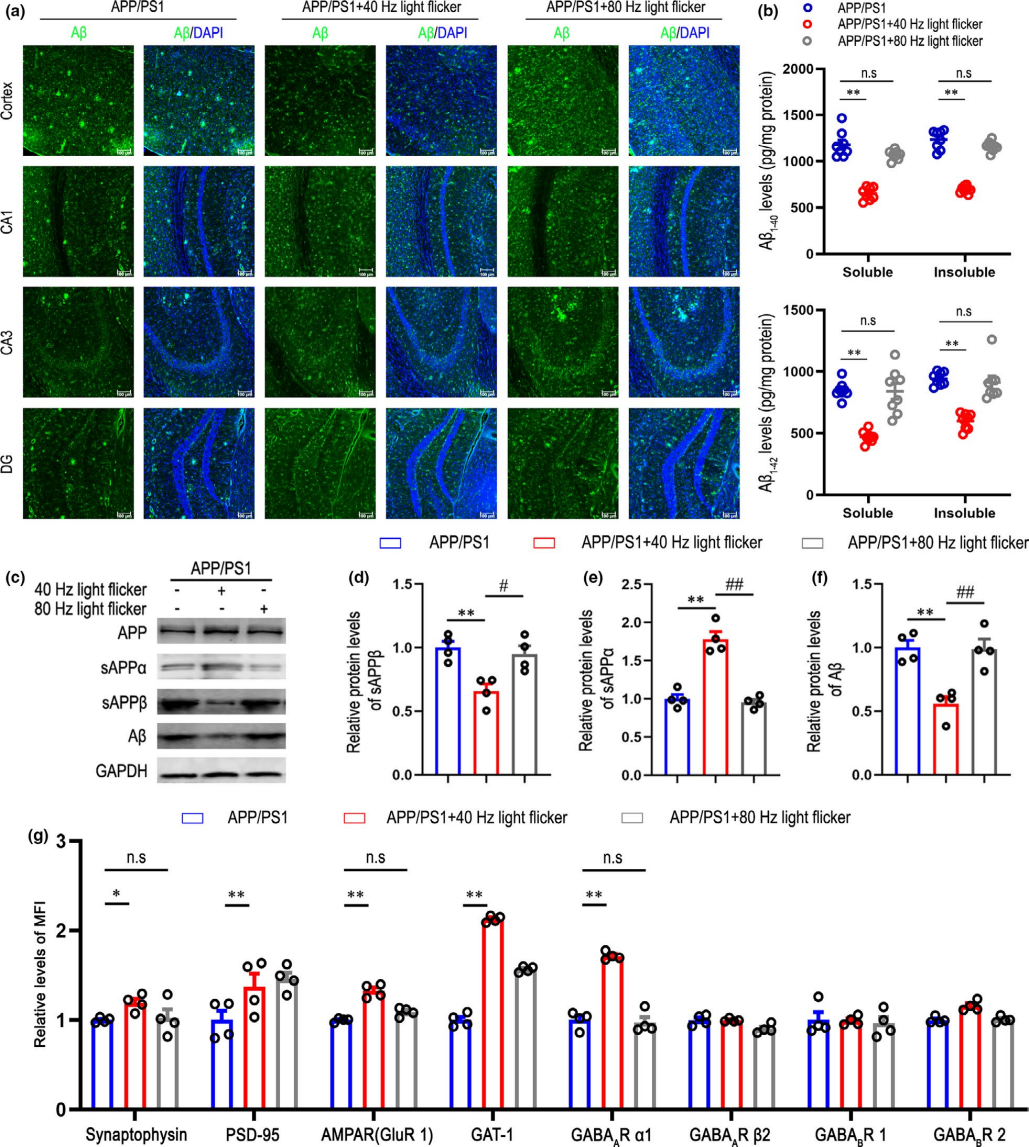
Gamma frequency light flicker reduces amyloid load in APP/PS1 mice. (a) Immunohistochemistry with anti‐Aβ (6E10, green) in cortex, CA1, CA3, and DG regions of 6‐month‐old male APP/PS1 mice following dark, 40 Hz, 80 Hz light flicker for 1 h/day for 7 days, scale bar represents 100 μm, *n* = 7 mice/group. DAPI labeling of cell nuclei (blue). (b) Soluble and insoluble Aβ_1‐40_ and Aβ_1‐42_ levels in cortex of 6‐month‐old male APP/PS1 mice after 7 days of 1 h per day dark, 40 Hz, or 80 Hz flicker were performed by ELISA (*n* = 8 mice per group). Data are presented as mean ± SEM. *p < 0.05 vs. APP/PS1 group, **p < 0.01 vs. APP/PS1 group, *n*.*s* = not significant, by two‐way ANOVA with *Tukey's post hoc* multiple comparisons test. (c) Representative Western blot showing levels of APP, sAPPα, sAPPβ, and Aβ in cerebral cortex of 6‐month‐old male APP/PS1 mice with dark, 40 Hz, or 80 Hz light flicker (*n* = 4 mice per group). Data are presented as mean ± SEM. *p < 0.05 vs. APP/PS1 group, **p < 0.01 vs. APP/PS1 group, *#p* < 0.05 vs. indicated group, *##p* < 0.01 vs. indicated group, by two‐way ANOVA with *Tukey's post hoc* multiple comparisons test. (d) Relative immunoreactivity of sAPPβ normalized to GAPDH. (e) Relative immunoreactivity of sAPPα normalized to GAPDH. (f) Relative immunoreactivity of Aβ normalized to GAPDH. (g) The expression levels of synaptophysin, PSD‐95, AMPAR (GluR1), GAT‐1, GABA_A_R α1, GABA_A_R β2, GABA_B_R1, and GABA_B_R2 were detected by flow cytometry in different groups (*n* = 4 mice per group). MFI: mean fluorescence intensity. Data are presented as mean ± SEM. *p < 0.05 vs. APP/PS1 group, **p < 0.01 vs. APP/PS1 group, *n*.*s* = not significant, by two‐way ANOVA with *Tukey's post hoc* multiple comparisons test

Aβ accumulation is thought to initiate multiple neurotoxic events that are typical of AD pathology (Selkoe et al., 1996). Therefore, we examined whether gamma frequency light flicker affected overall Aβ levels in APP/PS1 mice. Six‐month‐old APP/PS1 mice, which have amyloid‐plaque pathology in cortex and hippocampus, were placed in a dark chamber for 1 h daily over 7 days with 40 Hz light flicker (635 nm light pulse at 40 Hz), 80 Hz light flicker, or dark. As shown in Figure 1a, the intensity of Aβ labeling especially in cortex of APP/PS1 mice stimulated with 40 Hz light flicker was dramatically reduced. Additionally, biochemical analysis in Figure 1b and Figure S1a revealed consistent results in Figure 1a, that is, a dramatic reduction in soluble Aβ_1‐40_ and Aβ_1‐42_ in APP/PS1 mice after treatment with 7 days of 1 h 40 Hz flicker. Insoluble Aβ_1‐40_ and Aβ_1‐42_ levels were similarly reduced. Moreover, the effect was related to 40 Hz light flicker as 80 Hz light flicker did not significantly reduce Aβ levels compared with dark control (Figure 1a,b and Figure S1a).

The amyloid concentration in the brain depends on Aβ production from intracellular APP‐proteolytic processing and Aβ‐clearance rates. As a transmembrane protein, APP enters Golgi network from endoplasmic reticulum (ER), which is rich in Golgi network. Some of the APP located in Golgi apparatus can enter the secretory pathway through *trans*‐Golgi network (TGN) and then toward the PM (Yamazaki et al., 1996). In addition, soluble amyloid precursor protein α (sAPPα) is described to be mainly produced in this route. To elucidate whether 40 Hz light flicker reduces Aβ production, we examined its effects on APP cleavage by measuring levels of the cleavage intermediates of APP, sAPPα, soluble amyloid precursor protein β (sAPPβ), and Aβ in APP/PS1 male mice (Figure 1c). Using the sAPPβ and sAPPα as markers of amyloidogenic and non‐amyloidogenic pathway (Herrero‐Labrador et al., 2020), respectively, we found that stimulation with 40 Hz light flicker significantly reduced sAPPβ (Figure 1d) and Aβ (Figure 1f), but increased sAPPα (Figure 1e) and sAPPα/sAPPβ ratio (Figure S1g), compared with those of dark control and 80 Hz light flicker. We next determined if 40 Hz light flicker altered APP‐proteolytic processing in APP/PS1 female mice (Figure S1b). After 40 Hz stimulation, we also found reduced sAPPβ (Figure S1c) and Aβ (Figure S1e), but increased sAPPα (Figure S1d) and sAPPα/sAPPβ ratio (Figure S1f), while dark and 80 Hz flickering did not. To demonstrate that these effects extend beyond APP/PS1 mice, we examined the effect of 40 Hz light flicker in 3×Tg mice (Figure S6), another well‐validated AD model, and found significantly reduced soluble Aβ_1‐40_ and Aβ_1‐42_ levels, as well as insoluble Aβ_1‐40_ and Aβ_1‐42_ levels. However, this effect did not occur under 80 Hz stimulation (Figure S6a). Furthermore, consistent with our findings in APP/PS1 mice, we observed a significant increase in sAPPα levels and sAPPα/sAPPβ ratio, compared to dark control group. In addition, the effect was specific to 40 Hz flicker as 80 Hz flicker did not show significant changes compared with dark control. Taken together, these results identify a non‐invasive 40 Hz light flicker treatment with a profound effect on amyloidogenesis.

Next, we carried out flow cytometry for synaptic marker synaptophysin, PSD‐95, GluR1 (AMPA receptor subunits), GAT‐1, GABA_A_R α1, GABA_A_R β2, GABA_B_R1, GABA_B_R2 which are involved in synaptic transmission, GABAergic inhibition, synaptic plasticity, and learning and memory (El‐Husseini et al., 2000). We found that the relative mean fluorescence intensity (MFI) levels of synaptophysin, GluR1, and GABA_A_R α1 were increased in 40 Hz light flicker group compared to APP/PS1 group (Figure 1g), but not in 80 Hz flicker stimulation group. These results suggest that light flicker regulates synaptic proteins, as well as GABAergic transmission‐related proteins, leading us to speculate light flicker might modulate synaptic connectivity, and synaptic functions. Moreover, as described in prior studies (Iaccarino et al., 2016), we found that 40 Hz flickering did increase power at 40 Hz, but 80 Hz stimulation did not (Figure S1h). Collectively, 40 Hz light flicker reduced Aβ load and thus may provide neuroprotective effects in AD.

### 2.2 Gamma frequency light flicker promotes APP anchoring to the plasma membrane for non‐amyloidogenic processing

**FIGURE 2:**
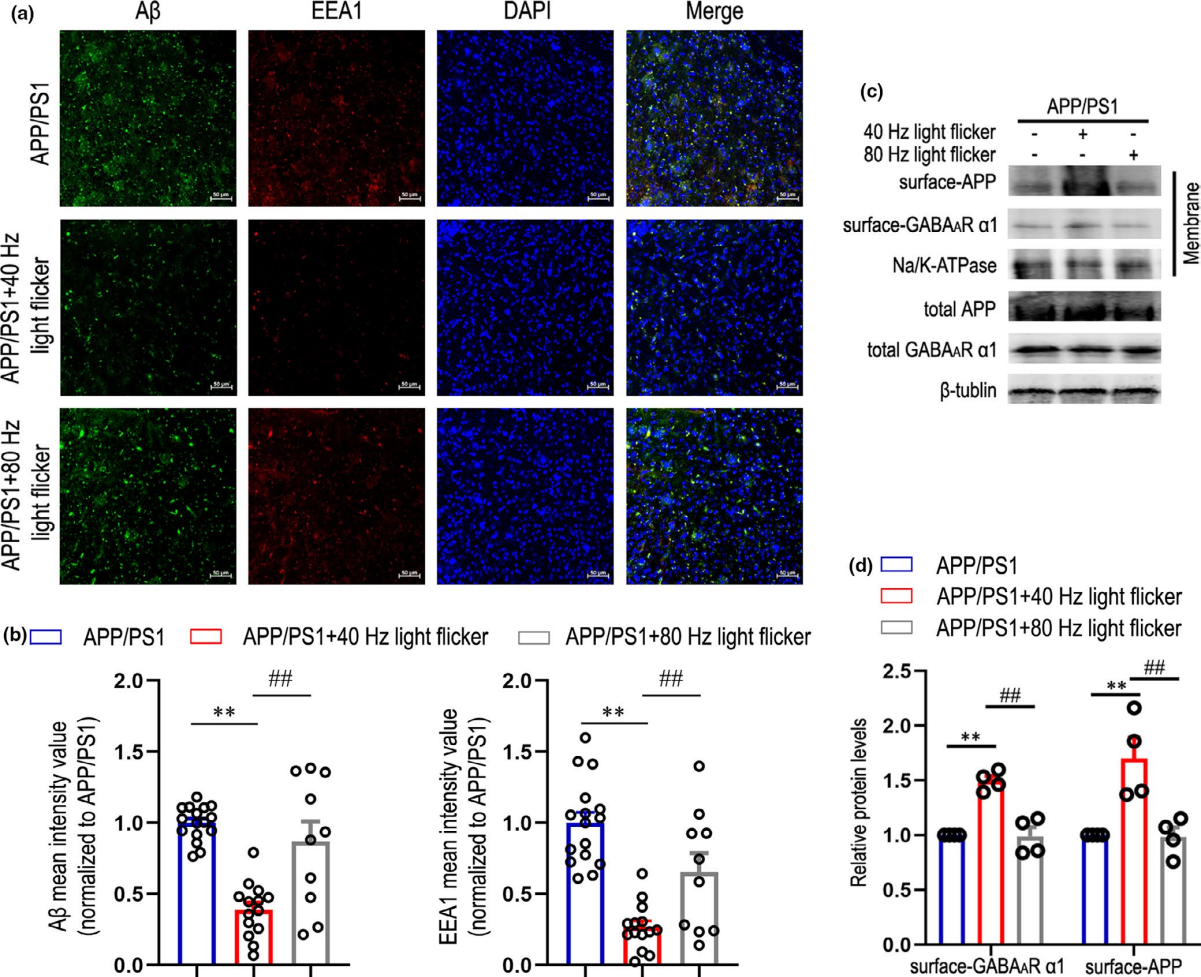
Gamma frequency light flicker promotes APP anchoring to the plasma membrane for non‐amyloidogenic processing in APP/PS1 mice. (a) Immunohistochemistry with anti‐Aβ (6E10, green) and EEA1 (red) in cortex of each group, scale bar: 50 μm. (b) Quantification of Aβ levels and EEA1 levels in cortex of different groups. Aβ or EEA1 mean fluorescence was analyzed by Image J software (*n* = 10–15 slices from five mice per group). Data are presented as mean ± SEM. *p < 0.05 vs. APP/PS1 group, **p < 0.01 vs. APP/PS1 group, *##p* < 0.01 vs. indicated group, by one‐way ANOVA with *Tukey's post hoc* multiple comparisons test. (c) Representative immunoblots of surface APP and GABA_A_R α1 levels in APP/PS1 mice under dark, 40 Hz, or 80 Hz light flicker. (d) Quantification of surface‐APP levels and surface‐GABA_A_R α1 levels (*n* = 4 mice per group). Data are presented as mean ± SEM. *p < 0.05 vs. APP/PS1 group, **p < 0.01 vs. APP/PS1 group, *#p* < 0.05 vs. indicated group, *##p* < 0.01 vs. indicated group, by two‐way ANOVA with *Tukey's post hoc* multiple comparisons test

Along the secretory pathway, a previous study showed that endosomes or the TGN as the major Aβ‐producing organelle, whereas the PM has been demonstrated as the predominant site for non‐amyloidogenic processing of APP by α‐secretase (Burgos et al., 2010; Choy et al., 2012; Lammich et al., 1999; Siman & Velji, 2003). Therefore, immunofluorescent staining with early endosomal antigen 1 (EEA1) to labeling endosomes in cortex of APP/PS1 mice after 40 Hz light flicker was significantly lower than that of the dark control group and 80 Hz flicker stimulation group (Figure 2a,b). Additionally, immunostaining with Aβ in cortex after 40 Hz light flicker was consistent with the results in Figure 1a–c. These results suggest that, in addition to the observed changes in APP cleavage products, 40 Hz flicker may also alter APP endosomal processing. To further explore whether 40 Hz light flicker can inhibit BACE1 cleavage localized in endosomes by promoting APP anchoring to the PM, we performed plasma membrane protein isolation with reference to the previously described experimental method (Kutluay et al., 2014; Lin et al., 2016) to detect the levels of full‐length APP and GABA_A_R α1 in different groups. The results showed that compared with dark control, APP/PS1 mice treated with 40 Hz flicker had remarkably increased surface‐APP and surface‐GABA_A_R α1, while 80 Hz light flicker did not (Figure 2c,d). In addition, we obtained similar results in female APP/PS1 mice (Figure S1i). We also performed plasma membrane protein isolation in 3×Tg mice (Figure S6c), consistent with our observations in APP/PS1 mice, we found a significant increase in surface‐APP and surface‐GABA_A_R α1 levels after 40 Hz light flicker compared with dark control. However, this effect does not occur under 80 Hz stimulation. All of our present results suggested that 40 Hz light flicker potentially enhanced APP anchoring to the PM for non‐amyloidogenic processing and competitively inhibited BACE1 cleavage, ultimately reducing Aβ production. It has been proposed that GABA_A_R‐mediated GABAergic neurotransmission is critical for the effects of 40 Hz flicker on Aβ levels (Iaccarino et al., 2016). However, the specific molecular mechanism of 40 Hz light flicker regulating GABA_A_R α1 distribution in the PM and whether the light‐flicker‐induced increase in GABA_A_R α1 levels on the PM further affects the GABAergic inhibitory effect, which is ultimately reflected in the regulation of gamma oscillations, still need further study.

### 2.3 APP‐KCC2 interaction is enhanced by gamma frequency light flicker to stabilize KCC2 on the plasma membrane

**FIGURE 3:**
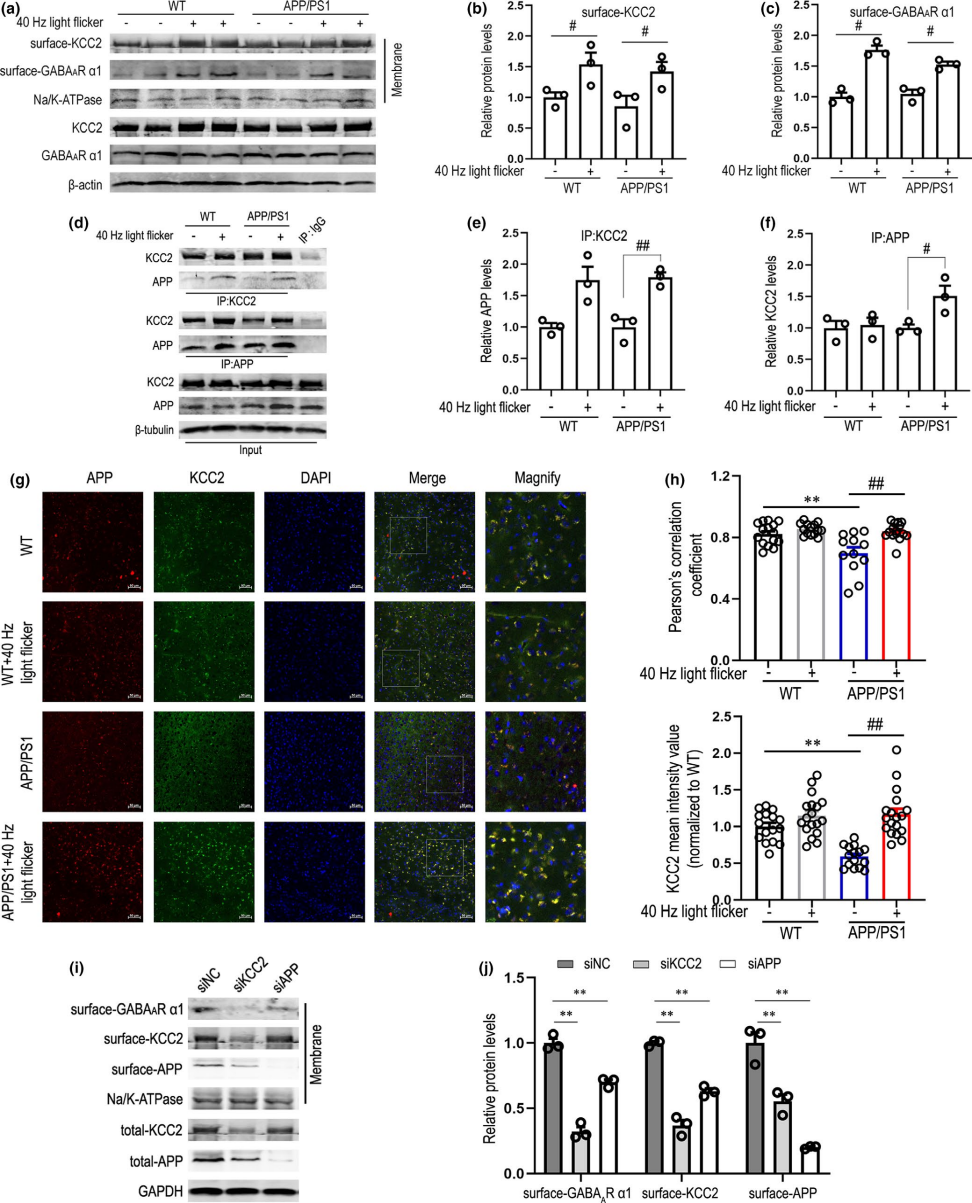
APP‐KCC2 interaction is enhanced by gamma frequency light flicker to stabilize KCC2 on the plasma membrane. (a) Representative immunoblots of surface KCC2 and GABA_A_R α1 levels in 6‐month‐old WT or APP/PS1 mice under 7 days of 1 h/day 40 Hz light flicker or not (*n* = 3 mice per group). Data are presented as mean ± SEM. #*p* < 0.05 vs. indicated group, by two‐way ANOVA with *Tukey's post hoc* multiple comparisons test. (b) Quantification of surface‐KCC2 levels. (c) Quantification of surface‐GABA_A_R α1 levels. (d) Representative Western blots showing co‐immunoprecipitation with both KCC2 and APP antibodies in cerebral cortex of 6‐month‐old WT or APP/PS1 mice with or without 40 Hz light flicker (*n* = 3 mice per group). Data are presented as mean ± SEM. #*p* < 0.05 vs. indicated group, ##*p* < 0.01 vs. indicated group, by unpaired *t*‐test. (e) Relative immunoreactivity of APP normalized to KCC2 (IP: KCC2). (f) Relative immunoreactivity of KCC2 normalized to APP (IP: APP). (g) Immunohistochemistry with anti‐APP (red) and KCC2 (green) in cerebral cortex of 6‐month‐old WT or APP/PS1 under 7 days of 1 h/day 40 Hz light flicker or not. Scale bar, 50 μm. (h) Pearson's correlation coefficient analysis of APP and KCC2, and quantification of KCC2 levels in different groups (*n* = 18 slices from 7 to 9 mice per group). Data are presented as mean ± SEM. *p < 0.05 vs. WT group, **p < 0.01 vs. WT group; *#p* < 0.05 vs. indicated group, *##p* < 0.01 vs. indicated group, by two‐way ANOVA with *Tukey's post hoc* multiple comparisons test. (i) Representative immunoblots of surface KCC2, GABA_A_R α1, and APP levels in siNC, siKCC2, and siAPP treatment group. (j) Quantification of surface‐KCC2, surface‐GABA_A_R α1, surface‐APP levels (*n* = 3). Data are presented as mean ± SEM. *p < 0.05 vs. control group, **p < 0.01 vs. control group; *#p* < 0.05 vs. indicated group, *##p* < 0.01 vs. indicated group, by two‐way ANOVA with *Tukey's post hoc* multiple comparisons test

The best‐characterized effectors of the Cl^−^ gradients in the central nervous system are KCC2 and the Na‐K‐2Cl cotransporter. KCC2 performs its function by transporting Cl^−^ in response to concentration gradients to ensure appropriate intracellular Cl^−^ concentration, which is essential for maintaining appropriate postsynaptic inhibition mediated by GABA_A_Rs (Ben‐Ari, 2002; Ben‐Ari et al., 2012; Boulenguez et al., 2010). KCC2 is mainly localized on the cell surface for its functioning (Gauvain et al., 2011). We next asked whether plasma membrane localization of KCC2 and GABA_A_R α1 were increased via treatment with 40 Hz light flicker. To do so, we isolated and measured plasma membrane protein levels in the cortex of WT and APP/PS1 mice with or without 40 Hz light flicker (Figure 3a). We observed significantly increased surface‐KCC2 levels in WT and APP/PS1 groups treated with 40 Hz flicker, respectively (Figure 3b), and surface‐GABA_A_R α1 levels were also similarly increased (Figure 3c). Previous studies have shown that APP deficiency results in significant reductions in both total and surface KCC2 levels, leading to a depolarizing shift in the GABA reversal potential (Chen et al., 2017). In this particular study, only full‐length, but not intracellular or extracellular fragments of APP could stabilize the normal total and surface KCC2 protein levels. Combined with the results in Figure 1d and Figure 2a, 40 Hz flicker inhibited APP endosomal processing, implying reduced the BACE1‐mediated cleavage, such that the full‐length APP anchoring within the PM could be increased. The results in Figure 2c also illustrate this speculation. To further determine whether 40 Hz light flicker regulates the interaction between APP and KCC2, we performed co‐immunoprecipitation experiments. As shown in Figure 3d, through detection of APP in anti‐KCC2 immunoprecipitates or KCC2 in anti‐APP immunoprecipitates, 40 Hz flicker enhanced the direct physical protein–protein interaction between APP and KCC2, especially in the APP/PS1 group. This effect was specific to 40 Hz light flicker in APP/PS1 male mice, as 80 Hz flicker stimulation significantly did not increase APP‐KCC2 interaction compared with dark control (Figure S2h–j), but in APP/PS1 female group, the detection of KCC2 in anti‐APP immunoprecipitates showed no significant change among groups. We also examined APP‐KCC2 interaction after 40 Hz light flicker in another AD mouse model (Figure S6d), and found a remarkable increase in the detection of APP in anti‐KCC2 immunoprecipitates versus dark control, while no significant change was observed in 80 Hz light flicker. In addition, we observed slight, non‐significant increase in the detection of KCC2 in anti‐APP immunoprecipitates after 40 Hz flicker versus control. Furthermore, immunofluorescent staining of APP and KCC2 showed that a very close interaction between APP and KCC2 was detected under the 40 Hz light flicker treatment (Figure 3g). Pearson's correlation coefficient analysis further showed that 40 Hz flicker increased the co‐localization of APP and KCC2 (Figure 3h). Interestingly, the fluorescent intensity of KCC2 in the APP/PS1 group was significantly lower than that in the WT group but 40 Hz flicker ameliorated this phenotype (Figure 3h). Furthermore, immunofluorescence of APP and GABA_A_R α1 showed that light flicker significantly enhanced the co‐localization of APP and GABA_A_R α1 (Figure S2a,b).

We next performed flow cytometry to determine the percentage numbers and MFI of surface‐GABA_A_R α1^+^ in APP^+^ cells. As shown in Figure S2c,d, 40 Hz light flicker significantly increased the percentage numbers and relative expression of surface‐GABA_A_R α1^+^ in APP^+^ cells. However, by detecting GABA_A_R α1 subunit in anti‐APP immunoprecipitates (Figure S2e), we did not observe a direct protein–protein interaction between APP and GABA_A_R α1 subunit. To further determine whether APP was responsible for maintaining normal total and surface KCC2 levels, RNA interference was used to silence APP or KCC2 and then total‐KCC2 and surface‐KCC2 were detected by plasma membrane protein isolation experiments. The results showed that silencing APP resulted in a reduction in total‐KCC2 levels (Figure 3i and Figure S2g), and even in surface‐KCC2 levels (Figure 3j). Notably, surface‐GABA_A_R α1 subunit levels were significantly reduced in siAPP or siKCC2 group. These results support a model in which loss of full‐length APP resulted in reduced KCC2 levels and decreased KCC2 functions, which causes the attenuation of GABA_A_R α1 subunit expression on the PM. Gamma frequency light flicker ensures that more full‐length APP is anchored on the PM to function, rather than being cleaved by BACE1 to produce Aβ. Therefore, total and surface levels of APP and KCC2 play an important role in maintaining surface‐GABA_A_R α1 subunit levels. Recent studies on KCC2 processing demonstrate that the intrinsic ion transport rate, cell surface stability, and trafficking of KCC2 are modulated by the phosphorylation of critical serine and tyrosine residues at the C‐terminus of this protein (Bergeron et al., 2006; Lee et al., 2007, 2010, 2011). Whether the increased surface stability of KCC2 induced by 40 Hz light flicker is through the regulation of its post‐translational modification process is poorly understood.

### 2.4 Gamma frequency light flicker suppresses KCC2 internalization and subsequent degradation via regulating both tyrosine phosphorylation and ubiquitination, leading to an increase in surface‐KCC2 levels

**FIGURE 4:**
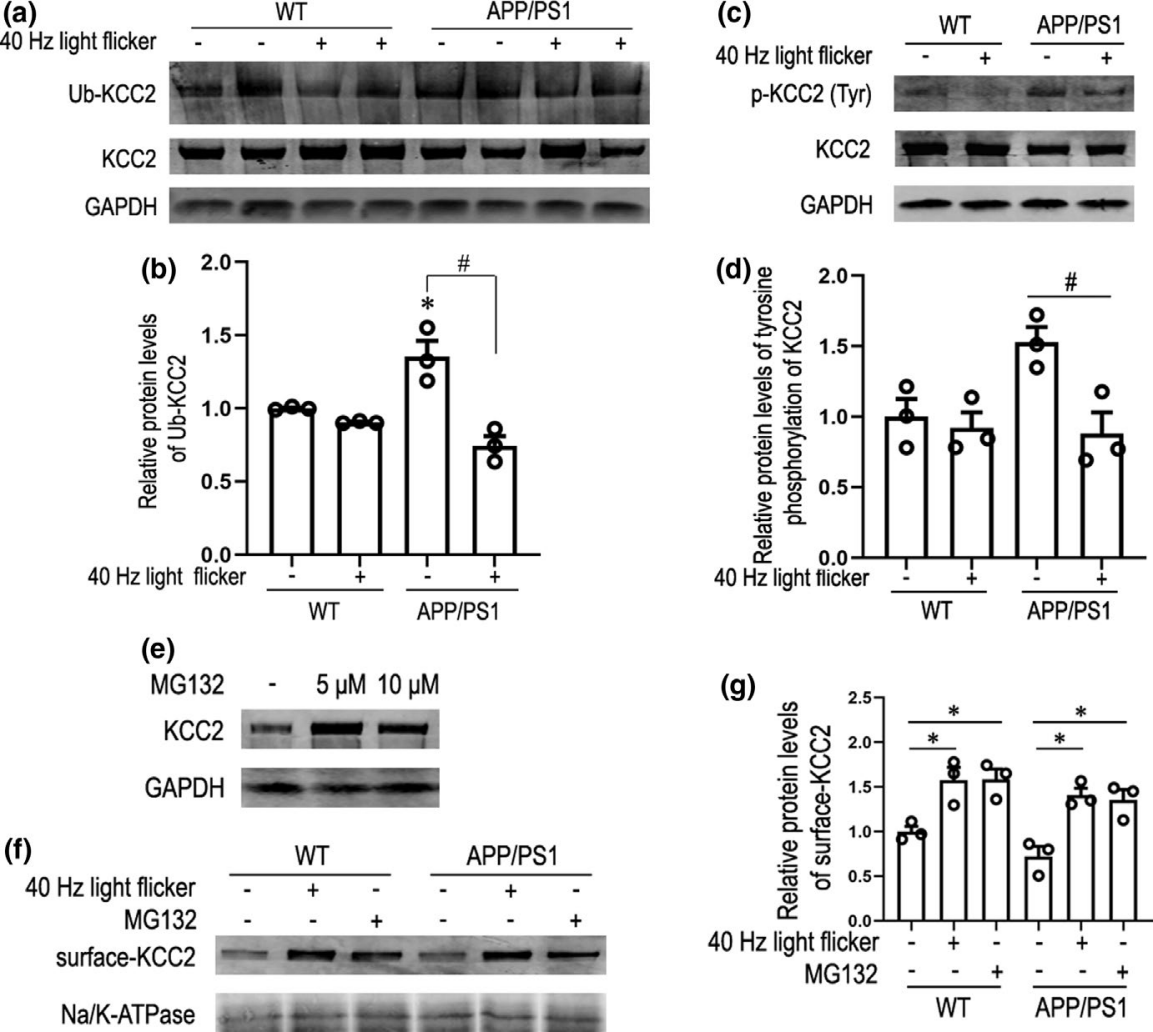
Gamma frequency light flicker suppresses KCC2 internalization and subsequent degradation via regulating both tyrosine phosphorylation and ubiquitination, leading to an increase in surface‐KCC2 levels. (a) Cortex extracted from 6‐month‐old WT and APP/PS1 littermates treated with 7 days of 1 h/day dark or 40 Hz light flicker immunoprecipitated with an anti‐KCC2 antibody (IP: KCC2) and probed with anti‐ubiquitin antibody. (b) Quantification of the ubiquitinated KCC2 (Ub‐KCC2) for each group (*n* = 3 mice per group). Data are presented as mean ± SEM. *p < 0.05 vs. WT group, *#p* < 0.05 vs. indicated group, by two‐way ANOVA with *Tukey's post hoc* multiple comparisons test. (c) Cortex isolated from 6‐month‐old WT and APP/PS1 littermates with or without 7 days of 1 h/day 40 Hz light flicker immunoprecipitated with an anti‐KCC2 antibody (IP: KCC2) and probed with anti‐phospho‐Tyrosine antibody. (d) Quantification of phosphorylated KCC2 on tyrosine (p‐KCC2 (Tyr)) for each group (*n* = 3 mice per group). Data are presented as mean ± SEM. *#p* < 0.05 vs. indicated group, by two‐way ANOVA with *Tukey's post hoc* multiple comparisons test. (e) Representative immunoblots of KCC2 incubated with MG132 at different concentrations. (f) Representative immunoblots of membrane proteins from 6‐month‐old WT or APP/PS1 mice treated with or without 7 days of 1 h/day 40 Hz light flicker and MG132. (g) Relative immunoreactivity of surface‐KCC2 normalized to Na/K‐ATPase (*n* = 3). Data are presented as mean ± SEM. *p < 0.05 vs. control group, by two‐way ANOVA with *Tukey's post hoc* multiple comparisons test

Given the critical role that KCC2 plays in neuronal function, there is considerable interest in how its functional expression is controlled. Much of the emphasis has been placed on the role of phosphorylation (Kahle et al., 2005; Lee et al., 2007; Miho et al., 2009; Wake et al., 2007). Tyrosine phosphorylation of KCC2 decreases the cell surface stability principally by enhancing its lysosomal degradation (Lee et al., 2011). Next, we examined whether levels of ubiquitination and tyrosine phosphorylation of KCC2 were decreased in the 40 Hz light flicker group. Cerebral cortex tissue from different groups was lysed and immunoprecipitated with an anti‐KCC2 antibody. Precipitates were blotted for ubiquitinated (Ub) and tyrosine phosphorylated proteins using an anti‐ubiquitin and anti‐P‐Tyr antibody, respectively. We observed a robust increase in Ub‐KCC2 levels in APP/PS1 mice compared to those of WT mice. However, there was a significant decrease in the levels of Ub‐KCC2 in 40 Hz light‐flicker‐stimulated APP/PS1 mice, compared to those of the dark control group (Figure 4a,b). In addition, it was found that immunoprecipitated KCC2 was phosphorylated at tyrosine residues in APP/PS1 mice (Figure 4c), but that 40 Hz flicker abated the effect. Additionally, this effect was specific to 40 Hz light flicker in APP/PS1 mice (Figure S3e), as 80 Hz flicker stimulation did not significantly decrease the levels of Ub‐KCC2 and p‐KCC2 (Tyr) compared with dark group. Next, incubating slices of APP/PS1 mice with the proteasome inhibitor, MG132, increased levels of KCC2 at concentrations of 5 and 10 μM (Figure 4e). Moreover, using MG132 as a positive control demonstrated that 40 Hz flicker inhibited ubiquitin–proteasome pathway of KCC2 (Figure S3a,b). Next, we further investigated whether limiting degradation of KCC2 contributed to the increase of surface‐KCC2 under gamma frequency light flicker treatment. The results showed that surface‐KCC2 levels were increased by MG132 in cortex of WT and APP/PS1 mice, as well as by 40 Hz flicker (Figure 4f,g). Together, these data indicate that treatment with 40 Hz light flicker reduces KCC2 internalization and subsequent degradation via inhibiting both tyrosine phosphorylation and ubiquitination, leading to an increase in surface‐KCC2 levels.

To further demonstrate that KCC2 instability is due, at least in part, to the association with APP, we test whether silencing APP leads to degradation of KCC2. Data in Figure S3d have shown that knocking down APP resulted in a decrease in KCC2 levels, while MG132 significantly improved its level. In addition, we tested whether blockade of tyrosine kinases by PP2, a potent inhibitor of Src‐family tyrosine kinases, enhances KCC2 levels even in the absence of APP. Treatment of cells transfected with siAPP with PP2 at a concentration of 20 μM significantly increased KCC2 levels, suggesting that reduction of full‐length APP leads to tyrosine phosphorylation and subsequent degradation of KCC2. This result led us to hypothesize that in AD model, the full‐length APP mostly produces Aβ through the β, γ‐secretase‐mediated cleavage pathway, while the full‐length APP level located on the PM is relatively low, but our experimental data indicate that the levels of full‐length APP located on the PM are significantly upregulated in APP/PS1 mice stimulated by 40 Hz light flicker, increasing its interaction with KCC2, which is likely to be involved in the post‐translational modification process of KCC2.

### 2.5 Activated PKC by gamma frequency light flicker phosphorylates APP and KCC2 to maintain plasma membrane levels of both, which contributes to the upregulation of surface‐GABA_A_R α1

**FIGURE 5:**
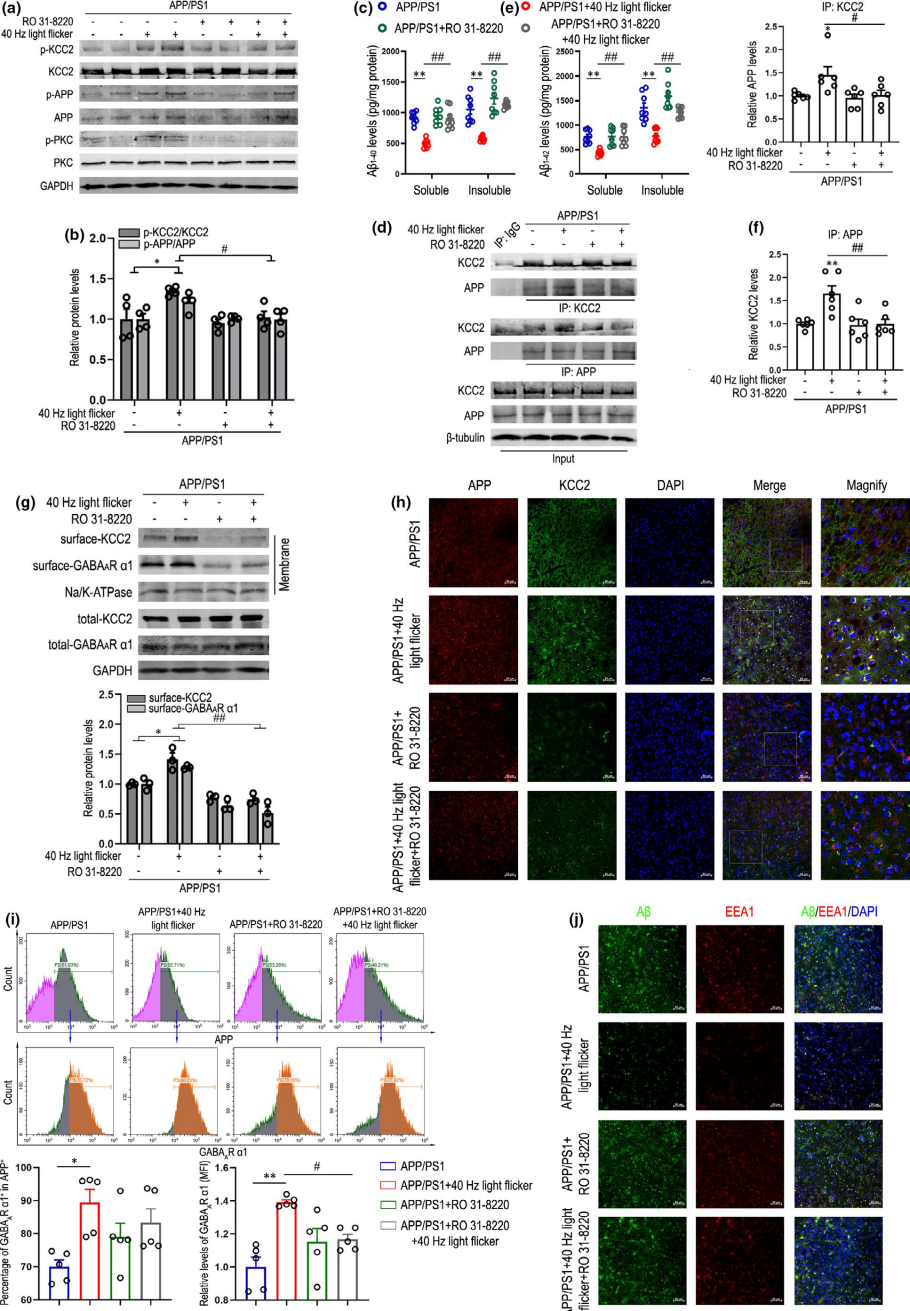
Activated PKC by gamma frequency light flicker phosphorylates APP and KCC2 to maintain membrane levels of both, which contributes to the upregulation of surface‐GABA_A_R α1. (a) Representative immunoblots showing levels of p‐PKC in cortex of 6‐month‐old APP/PS1 mice after 7 days of 1 h/day dark, 40 Hz flicker, RO 31‐8220 (6 mg/kg/d, s.c), RO 31‐8220 (6 mg/kg/d, s.c) with 40 Hz flicker. Immunoprecipitates were analyzed to detect the serine phosphorylation levels of APP and KCC2 with anti‐KCC2, anti‐APP, and anti‐phosphoserine antibodies. (b) Quantification of phosphorylated KCC2 and APP normalized to total KCC2 and APP (*n* = 4 mice per group). Data are presented as mean ± SEM. *p < 0.05 vs. APP/PS1 group; *#p* < 0.05 vs. indicated group; by two‐way ANOVA with *Tukey's post hoc* multiple comparisons test. (c) Soluble and insoluble Aβ_1‐40_ and Aβ_1‐42_ levels in cortex of APP/PS1 mice exposed to dark, 40 Hz flicker, RO 31‐8220 (6 mg/kg/d, s.c), RO 31‐8220 (6 mg/kg/d, s.c) with 40 Hz flicker were performed by ELISA (8 mice/group). Data are presented as mean ± SEM. **p < 0.01 vs. APP/PS1 group; *##p* < 0.01 vs. indicated group, by two‐way ANOVA with *Tukey's post hoc* multiple comparisons test. (d) Representative immunoblots showing co‐immunoprecipitation with both KCC2 and APP antibodies in cortex of APP/PS1 mice exposed to dark, 40 Hz flicker, RO 31‐8220 (6 mg/kg/d, s.c), RO 31‐8220 (6 mg/kg/d, s.c) with 40 Hz flicker (*n* = 6 mice/group). Data are presented as mean ± SEM. *p < 0.05 vs. APP/PS1 group; **p < 0.01 vs. APP/PS1 group; *#p* < 0.05 vs. indicated group; *##p* < 0.01 vs. indicated group, by two‐way ANOVA with *Tukey's post hoc* multiple comparisons test. (e) Relative immunoreactivity of APP normalized to KCC2 (IP: KCC2). (f) Relative immunoreactivity of KCC2 normalized to APP (IP: APP). (g) Representative immunoblots of membrane proteins from 6‐month‐old APP/PS1 mice exposed to 7 days of dark, 40 Hz flicker, RO 31‐8220 (6 mg/kg/d, s.c), RO 31‐8220 (6 mg/kg/d, s.c) with 40 Hz flicker (3 mice per group). Data are presented as mean ± SEM. *p < 0.05 vs. APP/PS1 group; *##p* < 0.01 vs. indicated group, by two‐way ANOVA with *Tukey's post hoc* multiple comparisons test. (h) Immunohistochemistry with anti‐APP (red) and anti‐KCC2 (green) in cortex of 6‐month‐old APP/PS1 treated with dark, 40 Hz light flicker, RO 31‐8220 (6 mg/kg/d, s.c), RO 31‐8220 (6 mg/kg/d, s.c) with 40 Hz flicker for 7 days (*n* = 5 mice/group). Scale bar, 50 μm. (i) Gates P2 (green gate) and P3 (orange gate) for surface APP and GABA_A_R α1 were determined, respectively, in the unstained group, and the number of APP^+^ cells (gate P2) was allowed to count 10,000 statistically in each experimental group, and the percentage number of GABA_A_R α1^+^ cells and mean fluorescence intensity (MFI) levels of surface GABA_A_R α1 in the gate P2 (APP^+^ cells) were analyzed on a CytoFLEX flow cytometer, using CytExpert software (*n* = 5 mice/group). Data are presented as mean ± SEM. *p < 0.05 vs. APP/PS1 group; **p < 0.01 vs. APP/PS1 group; *#p* < 0.05 vs. indicated group, by two‐way ANOVA with *Tukey's post hoc* multiple comparisons test. (j) Immunohistochemistry with anti‐Aβ (green) and anti‐EEA1 (red) antibodies in cortex of 6‐month‐old APP/PS1 treated with dark, 40 Hz light flicker, RO 31‐8220 (6 mg/kg/d, s.c), RO 31‐8220 (6 mg/kg/d, s.c) with 40 Hz flicker for 7 days (*n* = 6 to 7 mice per group), scale bar, 50 μm. DAPI labeling was used for cell nuclei

**FIGURE 6:**
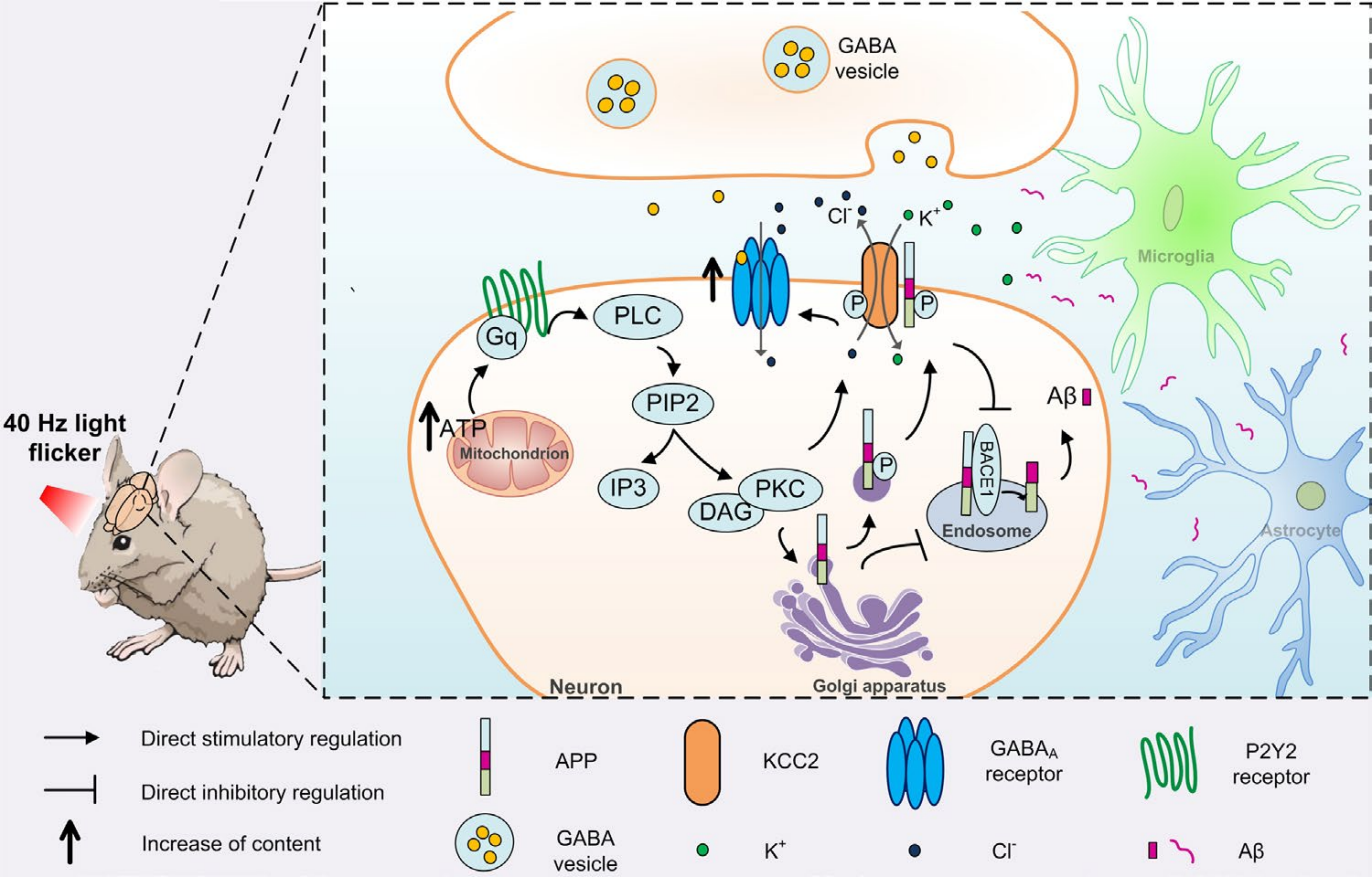
Model shows the potential mechanism by which 40 Hz light flicker reduces Aβ levels. Phosphorylation of APP induced by PKC activation under the treatment of 40 Hz light flicker led to maintained plasma membrane levels of full‐length APP as well as decreased trafficking to endosomes, which ultimately inhibited BACE1 cleavage pathway. Moreover, on the basis of PKC‐induced serine phosphorylation of KCC2, the tyrosine phosphorylation and degradation of KCC2 were further limited by a direct interaction with full‐length APP anchored within the plasma membrane, which contributed to the upregulation of surface GABA_A_ receptor α1 levels. In addition, the increase of ATP caused by 40 Hz light flicker promoted PLC/DAG signaling cascade, which is likely to be involved in the activation of PKC

The mechanisms underlying the actions of gamma frequency flicker on APP and KCC2 trafficking are currently unclear. One of the major mechanisms involved in the regulation of APP metabolism is phosphorylation, which influences APP subcellular trafficking and therefore influences intracellular APP‐proteolytic processing (da Cruz e Silva et al., 1995; Rebelo et al., 2007). The S655 residue lies within ^653^YTSI^656^ functional motif associated with APP traffic that can be phosphorylated by PKC, and leads to enhanced APP anterograde Golgi‐to‐PM trafficking (Ando et al., 2001; Icking et al., 2007; Vieira et al., 2009). Recent experiments on KCC2 processing suggest that phosphorylation of S940 mediated by PKC stabilizes KCC2 on the PM and increases cotransporter activity (Lee et al., 2007). To address the role of 40 Hz light flicker in regulating KCC2, we assessed whether KCC2 is directly phosphorylated at serine sites and whether this covalent modification alters transporter functional expression. Cerebral cortex tissue from different groups was lysed and immunoprecipitated with an anti‐KCC2 antibody. Precipitates were blotted for serine‐phosphorylated proteins using an anti‐phosphoserine antibody. Our studies demonstrated that KCC2 was phosphorylated at serine sites via 40 Hz flicker that depended on PKC activation (Figure 5a,b, and Figure S4a), thereby increasing the cell surface stability of KCC2 (Figure 5g), which provided a basic guarantee for the enhancement of APP‐KCC2 interaction under 40 Hz flicker treatment (Figure 5d,f). Moreover, we observed a robust increase in serine‐phosphorylated APP levels, which positively modulated PKC‐dependent APP secretory trafficking in the 40 Hz light‐flicker‐treated group (Figure 5a,b). As shown in Figure 5c, the robust reduction of total amyloid levels by 40 Hz flicker was likely mediated by enhancing APP anchoring to the PM: a PKC inhibitor, RO 31‐8220 (6 mg/kg/d, s.c), abolished this cytoprotective effect. Through detection of APP in anti‐KCC2 immunoprecipitates or KCC2 in anti‐APP immunoprecipitates, APP/PS1 mice treated with RO 31‐8220 resulted in a reduction of APP‐KCC2 interactions, even under 40 Hz light flicker (Figure 5d–f). It has been shown that expression of the surface GABA_A_R α1 subunit is regulated by surface‐KCC2 levels through affecting the intracellular Cl^−^ gradient. As shown in Figure 5g, RO 31‐8220 eliminated the increase of GABA_A_R α1 and KCC2 levels in the PM induced by 40 Hz flicker in APP/PS1 mice. Moreover, immunofluorescent staining with antibodies against APP and KCC2 (Figure 5h and Figure S4b) or APP and GABA_A_R α1 (Figure S4c) showed that inhibition of PKC by RO 31‐8220 even under 40 Hz flicker led to a reduction of co‐localized APP and KCC2 and an increase in the KCC2‐degradative process, which ultimately resulted in a decrease in APP‐GABA_A_R α1 co‐labeling. Similar results were obtained by flow cytometry. The results in Figure 5i showed that RO 31‐8220 eliminated the increases of the percentage numbers and relative expression levels of GABA_A_R α1^+^ in APP^+^ induced by 40 Hz light flicker in APP/PS1 mice. To further investigate whether 40 Hz light flicker inhibits endosomal processing of APP by activating PKC‐mediated signaling pathway, we performed immunofluorescent staining with antibodies against Aβ and EEA1. The results showed that 40 Hz light flicker prevented the increase in the co‐localization of Aβ with endosomes observed in the APP/PS1 group; however, RO 31‐8220 restrained this effect (Figure 5j and Figure S4d). Collectively, these results suggest a key role of PKC activation for 40 Hz light flicker to induce phosphorylation of APP and KCC2 in regulating both functional expressions (Figure 6).

Subsequently, we investigated the mechanism of PKC activation induced by light flicker. Activated phospholipase C (PLC) decomposes lipid inositol‐4,5‐diphosphate (PIP2) on the PM into diacylglycerol (DAG) and 1,4,5‐triphosphate inositol (IP3). IP3 binds with IP3 receptors located on the ER to release calcium into the cytoplasm. P2 receptors are a diverse family of plasma membrane proteins that can be segregated into two subtypes: the P2X receptors and the P2Y receptors. Activation of PLC by adenosine triphosphate (ATP)‐stimulated P2Y2 receptor leads to the generation of IP3 and DAG (Peterson et al., 2010). In addition, reactive oxygen species (ROS) can activate tyrosine‐protein kinases and lead to PLC activation (Kamata & Hirata, 1999), but can also induce DAG oxidation and lead to the activation of PKC (Kyung‐Mi et al., 2013). To uncover the molecular causes of PKC activation, we detected ATP, ROS, and calcium levels after 40 Hz light flicker in APP/PS1 mice. As shown in Figure S4a, combined with the results in Figure S5f, 40 Hz flicker significantly increased ATP levels, possibly by enhancing cytochrome c oxidase activity (Brookes et al., 2004; Wikström et al., 2018). Moreover, compared to that of the APP/PS1 group, 40 Hz flicker increased ROS levels (Figure S5b,c). However, a more detailed discussion of the mechanism involved still needs further study together.

## 3 DISCUSSION

In this study, we applied gamma frequency light flicker to relieve AD pathology in brain regions of the transgenic AD model with amyloid pathology. A key result of gamma frequency light flicker was the increase in the anchoring of APP to the PM, which competitively induced reduction of BACE1 cleavage. Additionally, we found that the full‐length APP anchored to the PM physically interacted with KCC2, which constituted an intracellular/extracellular Cl^−^ gradient and maintained surface GABA_A_R α1 subunit levels. More importantly, gamma frequency light flicker inhibited the phosphorylation of tyrosine sites and the ubiquitin–proteasome pathway of KCC2, but increased the phosphorylation of KCC2 at serine sites, which depended on the activation of PKC, thereby increasing the cell surface stability of KCC2, which provided a basic guarantee for the enhancement of APP‐KCC2 interaction under 40 Hz flicker treatment. Taken together, our observations demonstrated a non‐invasive approach to elicit system‐wide effects on AD‐related pathology.

Along the APP secretory pathway, the Golgi apparatus and the TGN have been demonstrated to be the major locations for Aβ production (Ayae et al., 2003). In contrast, the PM has been reported to be the predominant site for non‐amyloidogenic processing of APP (Lammich et al., 1999). A major finding from our present study was the regulation of APP trafficking by gamma frequency light flicker in these two transgenic mouse models of amyloidosis. A previous study showed that, in neurons, activation of PKC did not alter the expression of BACE1 but promoted BACE1 to translocate to the cell surface, where enzyme activity of BACE1 is relatively low (Das et al., 2013; Vassar et al., 2009; Wang et al., 2008). It has been known for some time that the aspartyl‐protease BACE‐1 is optimally active in an acidic pH (Vassar et al., 2009). Combined with our results in Figure 1c and Figure S1f‐g, we found that 40 Hz flicker indeed significantly increased sAPPα levels, as well as the sAPPα/sAPPβ ratios, and promoted APP anchoring to the PM. However, it remains unknown as to how 40 Hz flicker directly influences BACE1.

The results of siKCC2 experiments (Figure S7a) and GABA_A_ antagonist (picrotoxin, PTX, 0.18 mg/kg) treatment (Figure S7b) abrogated the effects of 40 Hz stimulation on Aβ levels suggest that the key role of KCC2 and GABAergic signals is necessary for this effect. Additionally, abundant evidence indicates that phosphorylation of KCC2 dynamically regulates its activity and cell surface expression (Rinehart et al., 2009). We hypothesized that pathophysiological levels of APP caused a reduction of surface‐KCC2 expression in APP/PS1 mice. In our current study, membrane‐separation experiments indicated that PKC‐dependent phosphorylation of KCC2 by 40 Hz light flicker promoted KCC2 stability on the cell surface and inhibited its degradation (Figures 3a–c and 5a–b). So how could 40 Hz light flicker affect surface‐KCC2 levels in WT group, which does not express human APP protein? Here, we employed an antibody to APP C‐terminal for Western blot to characterize APP trafficking by analyzing the C83/C99 ratio in WT mice. As shown in Figure S8c, compared with dark control, 40 Hz light flicker remarkably increased C83/C99 ratio in WT animals, indicating that 40 Hz stimulation promotes the non‐amyloidogenic pathway, and competitively inhibits BACE1‐mediated β‐secretase pathway. In addition, the results of plasma membrane isolation in Figure S8d showed that a significant increase in surface‐APP and surface‐GABA_A_R α1 levels after 40 Hz light flicker compared with dark control. Moreover, as shown in Figure S9, 40 Hz stimulation did not cause changes in KCC2 and GABA_A_R ɑ1 on the PM after knocking out APP. This result also suggested that the regulation of 40 Hz light flicker on the levels of surface‐KCC2 and surface‐GABA_A_R ɑ1 in WT should not be caused mainly by the increase of synaptogenesis, but more likely by anchoring endogenous APP on the PM, so as to ensure its interaction with KCC2. Based on our present findings, we propose a hypothesis that in the WT group, although human‐derived APP protein is not expressed, the murine derived full‐length APP protein expressed by itself interacts with KCC2 to promote their anchoring at the PM, and finally avoid APP cleavage by BACE1, and KCC2 degradation process, which contributes to the upregulation of surface‐GABA_A_R α1 levels, as shown in Figure 3. Taken together, our findings support the notion that such a non‐invasive drug‐free photobiomodulation treatment may represent a promising strategy to alleviate pathological changes associated with neurological disorders.

## 4 EXPERIMENTAL PROCEDURES

### 4.1 Resource and reagent

Reagent and resource used in this study were listed in Table 1.

**TABLE 1:** List of reagent or resource used in this study

Reagent or resource	Source	Identifier
*Antibodies*		
Anti‐GABA_A_ receptor α 1	Abcam	Cat# ab33299
Anti‐PKC	Abcam	Cat# ab23511
Anti‐GABA_B_ receptor 2	Abcam	Cat# ab75838
Anti‐GABA_A_ receptor β 2	Abcam	Cat# ab156000
Anti‐GABA_B_ receptor 1	Abcam	Cat# ab238130
AMPA Receptor (GluR 1) (D4N9V)	Cell Signaling Technology	Cat# 13185
Phospho‐PKC (pan) (βⅡ Ser660)	Cell Signaling Technology	Cat# 9371
KCC2	Cell Signaling Technology	Cat# 94725
EEA1	Cell Signaling Technology	Cat# 2411
Phospho‐Tyrosine	Cell Signaling Technology	Cat# 9411
APP	Cell Signaling Technology	Cat# 2452
β‐tubulin	Cell Signaling Technology	Cat# 86298
Anti‐APP C‐terminal	Merck Millipore	Cat# A8717
Anti‐Phosphoserine	Merck Millipore	Cat# 05‐1000
PSD95	Proteintech	Cat# 20665‐1‐AP
Ubiquitin	Proteintech	Cat# 10201‐2‐AP
Synaptophysin	Proteintech	Cat# 60191‐1‐lg
Na/K‐ATPase (ATP1A1)	Proteintech	Cat# 14418‐1‐AP
GAT1	Proteintech	Cat# 28488‐1‐AP
Beta‐Amyloid Monoclonal Antibody	Proteintech	Cat# 60342‐1‐lg
GAPDH	Santa Cruz Biotechnology	Cat# sc‐32233
β‐actin	Santa Cruz Biotechnology	Cat# sc‐47778
β‐amyloid	Santa Cruz Biotechnology	Cat# sc‐28365
Aβ (6E10)	Biolegend	Cat# 803015
sAPPα	Immuno‐Biological Laboratories	Cat# 11088
sAPPβ	Immuno‐Biological Laboratories	Cat# 10321
Goat Anti‐Mouse IgG H&L (Alexa Fluor 488)	Abcam	Cat# ab150113
Goat Anti‐Mouse IgG H&L (Alexa Fluor 555)	Abcam	Cat# ab150114
Goat Anti‐Rabbit IgG H&L (Alexa Fluor 488)	Abcam	Cat# ab150077
Goat Anti‐Mouse IgG H&L (Alexa Fluor 680)	Abcam	Cat# ab175775
Goat Anti‐Mouse IgG H&L (Alexa Fluor 790)	Abcam	Cat# ab175781
Goat Anti‐Rabbit IgG H&L (Alexa Fluor 647)	Abcam	Cat# ab150079
*Chemicals, peptides, plasmid*		
poly‐L lysine	Sigma	Cat# p6282
polyvinylidene difluoride (PVDF) membranes	Roche	Cat# 03010040001
DAPI (4’,6‐Diamidino‐2‐Phenylindole, Dihydrochloride)	Sigma	Cat# D9542
Fluo‐4 AM	Beyotime	Cat# S1060
ATP Assay Kit	Beyotime	Cat# S0026
Protein A+G Agarose	Beyotime	Cat# P2012
Reactive Oxygen Species Assay Kit	Beyotime	Cat# S0033
Human Aβ_1‐40_ and Aβ_1‐42_ ELISA kits	Invitrogen	Cat# KHB3481 Cat# KHB3442
MG132	MedChemExpress	Cat# HY‐13259
RO 31‐8220	MedChemExpress	Cat# HY‐13866A
Gö 6983	MedChemExpress	Cat# HY‐13689
Lipofectamine 3000	Invitrogen	Cat# L3000015
APP siRNA	Thermo Fisher Scientific	N/A
KCC2 siRNA	Santa Cruz Biotechnology	Cat# sc‐42607
Minute™ Plasma Membrane Protein Isolation and Cell Fractionation Kit	Invent Biotechnologies	Cat# SM‐005

### 4.2 Transgenic mice

The transgenic mice (APP/PS1) used in this study were produced by co‐injection with APPswe and PS1dE9 vectors (Jankowsky et al., 2003). All experimental mice were of a C57BL/6 background, and wild‐type and transgenic mice were paired from the litters and housed under the same living conditions. The triple‐transgenic model (3×Tg‐AD) harboring PS1(M146V), APPswe, and tau(P301L) transgenes (Oddo et al., 2003) were purchased from the Jackson Laboratory. The mice were housed in individual cages in a controlled environment (constant temperature 22 ± 1°C, humidity 50%–60%, lights on 07:00–19:00 h). Food and water were provided *ad libitum* unless otherwise noted. The experimenter was blind to animal genotypes, and no animals were excluded from analysis.

### 4.3 Light flicker stimulation protocol

For molecular and biochemical analyses, mice were then placed in a dark chamber illuminated by a light‐emitting diode (LED) bulb (635 nm) and exposed to following stimulation conditions: dark, 40 Hz flicker (12.5 ms light on, 12.5 ms light off, 47.9 W), 80 Hz flicker for 1 h. Fifteen minutes before the experiment, APP/PS1 mice were injected subcutaneously with saline (control) or RO 31‐8220 (6 mg/kg/d) (Hambleton et al., 2006). The APP/PS1 mice mentioned in the fifth part of the main text and in Figure 5 are the control mice treated with saline. However, due to the limitation of the number of words and the layout of the subgraph in figure, there is no additional annotation, which is explained here. Light flicker stimulation protocol was administered on daily basis for 1 h per day for the number of days as specified. Mice were allowed freely move inside the chamber but are not provided with food or water during the 1 h light flicker. After the light flicker exposure, mice were returned to their home cage and allowed to rest for further half an hour before being transported to the holding room (Adaikkan et al., 2019).

### 4.4 Western blot analysis and co‐immunoprecipitation

Western blot analysis and co‐immunoprecipitation (co‐IP) were performed following previous description with some modification (Bourdenx et al., 2021; Shen et al., 2021; Zhang et al., 2021). We performed plasma membrane protein isolation according to the directions provided by Minute™ Plasma Membrane Protein Isolation and Cell Fractionation Kit (Invent Biotechnologies, catalog number: SM‐005). The cerebral cortex (including visual cortex, somatosensory cortex, cingulate cortex, auditory cortex, and prefrontal cortex) was isolated from mice in different groups. See the Appendix S1 for details.

### 4.5 siRNA‐mediated gene silencing

See the Appendix S1 for details.

### 4.6 Immunohistochemistry

When collecting images from the cortical area of the immunohistochemistry experiment, each group will take four areas, namely the visual cortex, somatosensory cortex, cingulate cortex, and auditory cortex. See the Appendix S1 for details.

### 4.7 Enzyme‐linked immunosorbent assay for Aβ

The cerebral cortex (including visual cortex, somatosensory cortex, cingulate cortex, auditory cortex, and prefrontal cortex) was isolated from mice and subjected to Aβ measurement with the use of Aβ_1‐40_ or Aβ_1‐42_ Enzyme‐linked immunosorbent assay (ELISA) kit according to the manufacturer's instructions (Invitrogen, USA). The soluble Aβ fraction probably contained monomeric and oligomeric Aβ. Insoluble Aβ was treated with 5 M guanidine/50 mM Tris HCL (pH 8.0) buffer before ELISA measurement (Martorell et al., 2019).

### 4.8 Flow cytometry analysis

The cortex (including visual cortex, somatosensory cortex, cingulate cortex, auditory cortex, and prefrontal cortex) was collected from mice and washed with PBS. A single‐cell suspension was prepared through individual 70 μm cell strainer and washed in PBS as previous described (Mogilenko et al., 2021). Flow cytometry was used to analyze surface labeling of synaptophysin, PSD‐95, GluR1, GAT‐1, GABA_A_R α1, GABA_A_R β2, GABA_B_R1, GABA_B_R2 (Figure 1g). After fixation and blocking without permeabilization, the cells were stained with primary antibody for 1 h at room temperature and washed with PBS, and then, cells were stained with Alexa Fluor^®^ 488/647‐conjugated secondary antibodies, respectively. For surface receptor labeling (Figure S2c and Figure 5i), see the Appendix S1 for details.

### 4.9 Statistical analysis

Statistical analysis was conducted in SPSS software and GraphPad Prism 8. Data are expressed as the mean ± SEM. Significant differences were compared as noted in figure legends, using Student's *t*‐test for statistical analysis in two‐group comparison or one‐way/two‐way ANOVA with *Tukey's post hoc* multiple comparisons test for comparison among multiple groups, and the differences were considered statistically significant at *p* < 0.05. Specific statistical parameters are detailed in the figure legends.

## CONFLICT OF INTEREST

The authors declare that they have no competing interests.

## AUTHOR CONTRIBUTIONS

Q.S. and D.X. conceptualized and designed the project. Q.S., X.L.W., and Z.Z. performed stimulation experiments. Q.S., X.L.W., Z.Z., and D.Z. performed immunostainings and ATP analysis. Q.S., Z.Z., and D.Z. performed immunocoprecipitation and Western blotting. Q.S. and X.L.W. performed molecular biology experiments and ELISA. Q.S., X.L.W., and D.Z., performed flow cytometry. All the authors analyzed and interpreted the data. Q.S., S.H.Y., and D.X. wrote and revised the manuscript. S.H.Y. and D.X. provided the tools and supervised the project.

## Supporting information

Supplementary Material

## Data Availability

All data needed to evaluate the conclusions in the paper are available from the corresponding author upon reasonable request.
