# Supplementary material for: Gamma frequency light flicker regulates amyloid precursor protein trafficking for reducing β‐amyloid load in Alzheimer's disease model
Source: Aging Cell. 2022 Feb 23;21(3):e13573. doi: 10.1111/acel.13573 (PMC8920449; doi:10.1111/acel.13573)
Supplement: Supplementary file 1 — Supplementary Material [file ACEL-21-e13573-s001.docx]

Supplementary Information for

**Gamma frequency light flicker regulates APP trafficking for reducing Aβ load in Alzheimer's disease model**

Qi Shen^1,2^, Xiaolei Wu^1,2^, Zhan Zhang^1,2^, Di Zhang^1,2^, Sihua Yang^1,2*^, Da Xing^1,2,3*^

^1^MOE Key Laboratory of Laser Life Science & Institute of Laser Life Science, South China Normal University, Guangzhou 510631, China

^2^College of Biophotonics, South China Normal University, Guangzhou 510631, China

^3^Lead Contact

**^*^To whom correspondence and proof should be addressed**

Da Xing, Ph.D.

MOE Key Laboratory of Laser Life Science & Institute of Laser Life Science, College of Biophotonics, South China Normal University, Guangzhou 510631, China

E-mail: [xingda@scnu.edu.cn](mailto:xingda@scnu.edu.cn(()

Sihua Yang, Ph.D.

MOE Key Laboratory of Laser Life Science & Institute of Laser Life Science, College of Biophotonics, South China Normal University, Guangzhou 510631, China

E-mail: yangsh@scnu.edu.cn

**Materials and Methods**

**Animals**

APP knockout mice were purchased from the Jackson Laboratory. All of the procedures of animal experiments were approved by the Ethics Committee of South China Normal University and performed in accordance with the Association for Assessment and Accreditation of Laboratory Animal Care guidelines.

**Western blot analysis and co-immunoprecipitation**

For co-immunoprecipitation (co-IP), protein extracts were incubated with the indicated antibodies for 2-4 h at room temperature and then incubated with 50% slurry protein A-Sepharose at 4^o^C overnight. The pellet was resuspended with the same volume of SDS sample buffer and boiled 7 min to remove protein A-Sepharose beads. Then the whole cells lysates and immunoprecipitates were all analyzed by western blot analysis.

For plasma membrane protein isolation, according to the directions provided by Minute^TM^ Plasma Membrane Protein Isolation and Cell Fractionation Kit (Invent Biotechnologies, catalog number: SM-005), the brain tissues (including visual cortex, somatosensory cortex, cingulate cortex, auditory cortex and prefrontal cortex) were first sensitized by buffer A before passing through the proprietary filter in a zigzag manner when high-speed centrifugal force is applied, resulting in a cell lysate containing ruptured cell membranes and intact nuclei. As a result, the nuclear contaminations are virtually eliminated. Plasma membrane is further separated from the cell lysate (a mixture of crude membranes, intact nuclei, cytosol proteins and organelles) by subsequent differential and density centrifugation with a regular tabletop microcentrifuge. 5 distinct cell fractions (total membrane, plasma membrane, cytosol, nucleus and organelles) can be obtained at the completion of the protocol. Then obtained plasma membrane protein was analyzed by SDS-PAGE. And Na/K-ATPase was used as the plasma membrane marker ([Sato, Takahashi, Shigemoto-Mogami, Chujo, & Sekino, 2016](#_ENREF_5); [Zheng et al., 2016](#_ENREF_7)).

The cerebral cortex (including visual cortex, somatosensory cortex, cingulate cortex, auditory cortex and prefrontal cortex) was homogenized using RIPA (50 mM Tris HCl pH 8.0, 150 mM NaCl, 1% NP-40, 0.5% sodium deoxycholate, 0.1% SDS) buffer containing complete protease inhibitor cocktail (Roche), incubated on ice for 15 min, and rotated at 4 ^o^C for 30 min. Cell debris was isolated and discarded by centrifugation at 12,000 *r.p.m*. for 15 min. Lysates were quantitated by Bradford, and 60 μg protein was loaded on a 10% acrylamide gels. Protein was transferred from acrylamide gels to PVDF membranes (Roche) at 100V. Membranes were blocked using bovine serum albumin (5% w/v) diluted in TBST. Membranes were incubated in primary antibodies overnight at 4^o^C and followed by Alexa Fluor-conjugated secondary antibodies at room temperature for 2h. Detection was performed using the Odyssey infrared imaging system (Li-COR, Lincoln, NE, USA). The intensity of western blot signals was quantitated using ImageJ software.

**Immunohistochemistry**

For histological processing, mice were perfused with 4% paraformaldehyde (PFA) under deep anaesthesia, and the brains were post-fixed overnight in 4% PFA in 0.1 M phosphate buffer saline (PBS), then subsequently incubated in a sucrose series (10%, 20% and 30%) solution. Sequential coronal brain sections (15 μm thick) were obtained and mounted on polylysine-coated slides (Sigma-Aldrich). Sections were rinsed in PBS three times for 5 min and processed for antigen retrieval by boiling in 10 mM citrate buffer (pH 6.0) for 8 min. Sections were permeabilized and blocked in PBS containing 0.3% Triton X-100 and 5% bovine serum albumin (BSA) at room temperature for 1 h. Sections were incubated overnight at 4 ^o^C in primary antibody in PBS with 0.3% Triton X-100 and 5% BSA. Primary antibodies were detected with Alexa Fluor 488/555/647-conjugated secondary antibodies, and cell nuclei visualized with DAPI. To confirm ELISA experiments, the anti-Aβ antibody was used because it allowed for co-labelling with EEA1 and the anti-Aβ antibody (6E10) was used because it does not react with APP, allowing us to determine whether our labelling was specific to Aβ. Anti-APP antibody was used to co-labeling with KCC2 or GABA_A_R α1. Images were acquired using LSM 880 confocal microscopes (Zeiss) with 10 ×, 20 × or 40 × objectives at identical settings for all conditions, and quantified using ImageJ by an experimenter blind to treatment groups. For each experimental condition, two coronal sections per mouse from the indicated number of animals were used. And two to four images per mouse was used for quantification. In addition, the colocalization threshold plugin was used to measure co-localization of APP and KCC2, as well as APP and GABA_A_R α1.

**Electrophysiology recordings**

The mice were anaesthetized with isoflurane breathing anesthesia machine (ABM-100, Shanghai Yuyan Scientific Instruments Co., Ltd.), fixed on the stereotaxic brain locator (SA-102, Shanghai Yuyan Scientific Instruments Co., Ltd.), drilled and inserted into the electrode. The electrode is fixed with dental bracket powder to prevent the electrode from falling off. Open BL-420S biological function experiment system (Chengdu Taimeng Software Co., Ltd.), select electroencephalogram (EEG) detection experiment items, set parameters: time constant 0.3 s, filter 200 Hz. The EEG of mice was detected for more than 30 minutes. The average power spectral density of visual cortex was computed for each animal (within the same recording depth) over these trials ([Martorell et al., 2019](#_ENREF_4)).

**siRNA-mediated gene silencing**

A small interfering RNA (siRNA) plasmid targeted toward KCC2, and a nonsilencing control plasmid were both purchased from Santa Cruz Biotechnology. APP siRNA was purchased from Thermo Fisher Scientific. Lipofectamine 3000 was used to transfect constructs into cultured neurons with reference to the previously described experimental method ([Ivakine et al., 2013](#_ENREF_2); [Zhang, Shen, Wu, Zhang, & Xing, 2020](#_ENREF_6)), according to the manufacturer's instructions. For delivery of siRNA into neuronal culture, cells at 4 DIV were incubated with siRNA for APP, KCC2, or negative control (NC) with siRNA delivery media for 3 days ([Jiang et al., 2019](#_ENREF_3)).

**Real-time PCR**

Total RNA was extracted with RNAiso Plus (TaKaRa, D9108A) following the manufacturer's instructions. The first-strand cDNA was synthesized with a ReverTra Ace qPCR (Quantitative Real-time PCR) RT Kit (TOYOBO, FSQ-301). Primers for amplification of genes used in experiments were provided in figure legend. Real-time PCR was performed using SYBR Green PCR mix on a CFX Connect^TM^ Real-Time System normalized against GAPDH, and fold changes were calculated based on the 2^-△△Ct^ algorithm.

**Flow cytometry analysis**

For surface receptors labeling (Figure S2C and Figure 5I), cells were fixed with 4% PFA for 10 min and blocked with 3% BSA for 1 h at room temperature without permeabilization. The cells were stained with GABA_A_R α1 antibody at room temperature for 1 h, and washed three times with PBS. Then cells were stained with the Alexa Fluor® 647-conjugated Goat Anti-rabbit IgG secondary antibody at room temperature for 30 min in the dark. After PBS washing, the cells were stained with APP antibody at room temperature for 1 h, and washed three times with PBS. And then cells were stained with the Alexa Fluor® 488-conjugated Goat Anti-mouse IgG secondary antibody at room temperature for 30 min in the dark. For details, gates P2 and P3 for APP and GABA_A_R α1 were determined respectively in the unstained group, and the number of APP-positive (APP^+^) cells in the gate P2 was allowed to count 10000 statistically in each experimental group, and the percentage number of GABA_A_R α1-positive (GABA_A_R α1^+^) cells and relative mean fluorescence intensity (MFI) levels of surface GABA_A_R α1 in the gate P2 (APP^+^ cells) were analyzed on a CytoFLEX flow cytometer, using CytExpert software.

**Measurement of ATP levels**

The cerebral cortex (including visual cortex, somatosensory cortex, cingulate cortex, auditory cortex and prefrontal cortex) was homogenized using lysis buffer, incubated on ice for 15 min and then centrifugated at 12,000 *r.p.m*. at 4^o^C for 15 min. The supernatant was collected and then tested ATP with the use of ATP Assay Kit according to the manufacturer's instructions. A full ATP standard curve (1 nM-10 μM) was run in parallel in order to allow for extrapolation of ATP content in the experimental samples. ATP analysis was done based on luciferin/luciferase luminescence assay ([De et al., 2011](#_ENREF_1)).

**Ca^2+^, ROS and cytochrome c oxidase activity analysis**

To examine intracellular Ca^2+^ level, calcium-free buffers were used for tissue isolation and subsequent staining steps. The cerebral cortex (including visual cortex, somatosensory cortex, cingulate cortex, auditory cortex and prefrontal cortex) was isolated from mice and grinded with a homogenizer, resuspended with calcium-free buffers, and incubated with Fluo-4 AM at 37^o^C for 30 minutes. Flow cytometry was performed on a BD FACSCanto II flow cytometer (Becton Dickinson).

For ROS analysis, the cerebral cortex (including visual cortex, somatosensory cortex, cingulate cortex, auditory cortex and prefrontal cortex) was isolated from mice and grinded with a homogenizer, resuspended with PBS, and rotationally incubated with 2,7-Dichlorodi-hydrofluorescein diacetate (DCFH-DA) at 37^o^C for 30 minutes. Flow cytometry was performed on a BD FACSCanto II flow cytometer (Becton Dickinson).

For cytochrome c oxidase activity analysis, the cerebral cortex (including visual cortex, somatosensory cortex, cingulate cortex, auditory cortex and prefrontal cortex) was homogenized using lysis buffer, incubated on ice for 30 min and then centrifugated at 12,000 *r.p.m*. at 4^o^C for 15 min. The supernatant was collected and then tested the cytochrome c oxidase activity using the cytochrome c oxidase activity assay kit (Shanghai Enzyme-linked Biotechnology Co., Ltd., catalog number: ml037420) according to the manufacturer's instructions.

**Statistical analysis**

Statistical analysis was conducted in SPSS software and GraphPad Prism 8. Data are from one representative experiment among at least three independent experiments and are expressed as the mean ± SEM. Significant differences were compared as noted in figure legends, using Student's *t* test for statistical analysis in two-group comparison or one-way/two-way ANOVA with *Tukey's post hoc* multiple comparisons test for comparison among multiple groups, and the differences were considered statistically significant at *p <* 0.05.

**References**

De, T. L., Yu, J., El-Amouri, S., Gattoni-Celli, S., Richieri, S., Mccarthy, T., . . . Kindy, M. S. (2011). Transcranial laser therapy attenuates amyloid-β peptide neuropathology in amyloid-β protein precursor transgenic mice. *Journal of Alzheimers Disease, 23*(3), 521. doi:10.3233/JAD-2010-100894

Ivakine, E. A., Acton, B. A., Mahadevan, V., Ormond, J., Tang, M., Pressey, J. C., . . . McInnes, R. R. (2013). Neto2 is a KCC2 interacting protein required for neuronal Cl^−^ regulation in hippocampal neurons. *Proceedings of the National Academy of Sciences, 110*(9), 3561-3566. doi:10.1073/pnas.1212907110

Jiang, Y., Sato, Y., Im, E., Berg, M., Bordi, M., Darji, S., . . . Nixon, R. A. (2019). Lysosomal Dysfunction in Down Syndrome Is APP-Dependent and Mediated by APP-βCTF (C99). *The Journal of Neuroscience, 39*(27), 5255. doi:10.1523/JNEUROSCI.0578-19.2019

Martorell, A. J., Paulson, A. L., Suk, H.-J., Abdurrob, F., Drummond, G. T., Guan, W., . . . Tsai, L.-H. (2019). Multi-sensory Gamma Stimulation Ameliorates Alzheimer's-Associated Pathology and Improves Cognition. *Cell, 177*(2), 256-271. doi:10.1016/j.cell.2019.02.014

Sato, K., Takahashi, K., Shigemoto-Mogami, Y., Chujo, K., & Sekino, Y. (2016). Glypican 6 Enhances N-Methyl-D-Aspartate Receptor Function in Human-Induced Pluripotent Stem Cell-Derived Neurons. *Frontiers in Cellular Neuroscience, 10*(91), 259. doi:10.3389/fncel.2016.00259

Zhang, Z., Shen, Q., Wu, X., Zhang, D., & Xing, D. (2020). Activation of PKA/SIRT1 signaling pathway by photobiomodulation therapy reduces Aβ levels in Alzheimer's disease models. *Aging Cell, 19*(1), e13054. doi:10.1111/acel.13054

Zheng, W., Yang, J., Beauchamp, E., Cai, R., Hussein, S., Hofmann, L., . . . Tang, J. (2016). Regulation of TRPP3 channel function by N-terminal domain palmitoylation and phosphorylation. *Journal of Biological Chemistry, 291*(49), 25678-25691. doi:10.1074/jbc.M116.756544


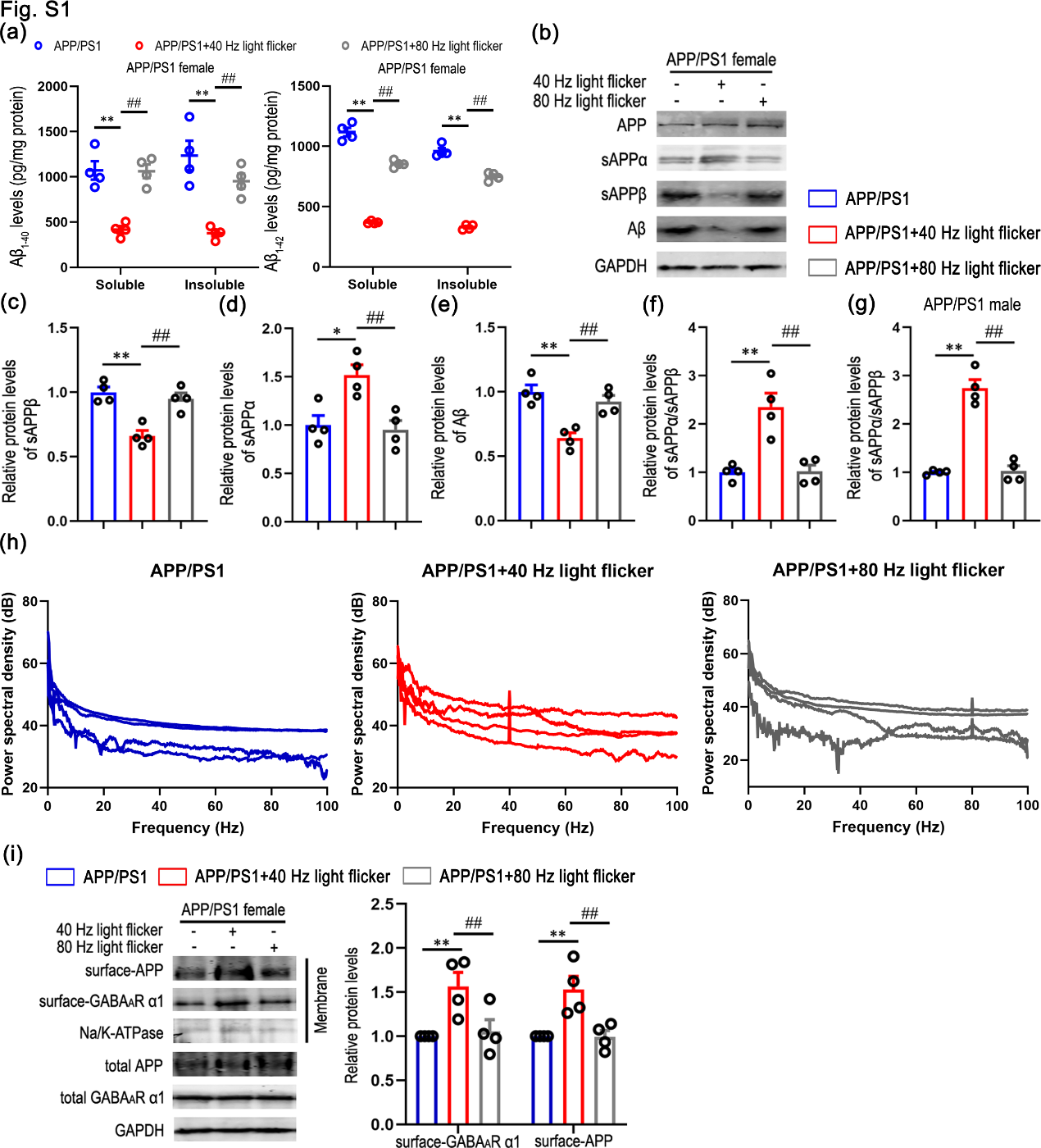


**Figure S1:** Gamma frequency light flicker ameliorates pathological burden in APP/PS1 mice. (a) Soluble and insoluble Aβ_1-40_ and Aβ_1-42_ levels in the cerebral cortex of 6-month-old female APP/PS1 mice following dark, 40 Hz, 80 Hz light flicker for 1 h/day for 7 days were performed by ELISA (*n* = 4 mice per group). Data are presented as mean ± SEM. **p* < 0.05 vs. APP/PS1 group, ***p* < 0.01 vs. APP/PS1 group, *#p* < 0.05 vs. indicated group, *##p* < 0.01 vs. indicated group, by two-way ANOVA with *Tukey's post hoc* multiple comparisons test. (b) Representative western blot showing levels of APP, sAPPα, sAPPβ and Aβ in cerebral cortex of 6-month-old male APP/PS1 mice with 7 days of 1 h/day dark, 40 Hz, or 80 Hz light flicker (*n* = 4 mice per group). Data are presented as mean ± SEM. **p* < 0.05 vs. APP/PS1 group, ***p* < 0.01 vs. APP/PS1 group, *#p* < 0.05 vs. indicated group, *##p* < 0.01 vs. indicated group, by two-way ANOVA with *Tukey's post hoc* multiple comparisons test. (c) Relative immunoreactivity of sAPPβ normalized to GAPDH. (d) Relative immunoreactivity of sAPPα normalized to GAPDH. (e) Relative immunoreactivity of Aβ normalized to GAPDH. (f) Relative sAPPα/sAPPβ ratios in APP/PS1 female group following dark, 40 Hz, or 80 Hz flicker stimulation. (g) Relative sAPPα/sAPPβ ratios in APP/PS1 male group following dark, 40 Hz, or 80 Hz flicker stimulation. (h) Power spectral density in visual cortex of APP/PS1 group response to dark, 40 Hz, or 80 Hz flicker stimulation (*n* = 4 mice per group). (i) Representative immunoblots and quantification of surface APP and GABA_A_R α1 levels in APP/PS1 female mice under 7 days of 1 h/day dark, 40 Hz, or 80 Hz light flicker (*n* = 4 mice per group). Data are presented as mean ± SEM. **p* < 0.05 vs. APP/PS1 group, ***p* < 0.01 vs. APP/PS1 group, *#p* < 0.05 vs. indicated group, *##p* < 0.01 vs. indicated group, by two-way ANOVA with *Tukey's post hoc* multiple comparisons test.

**
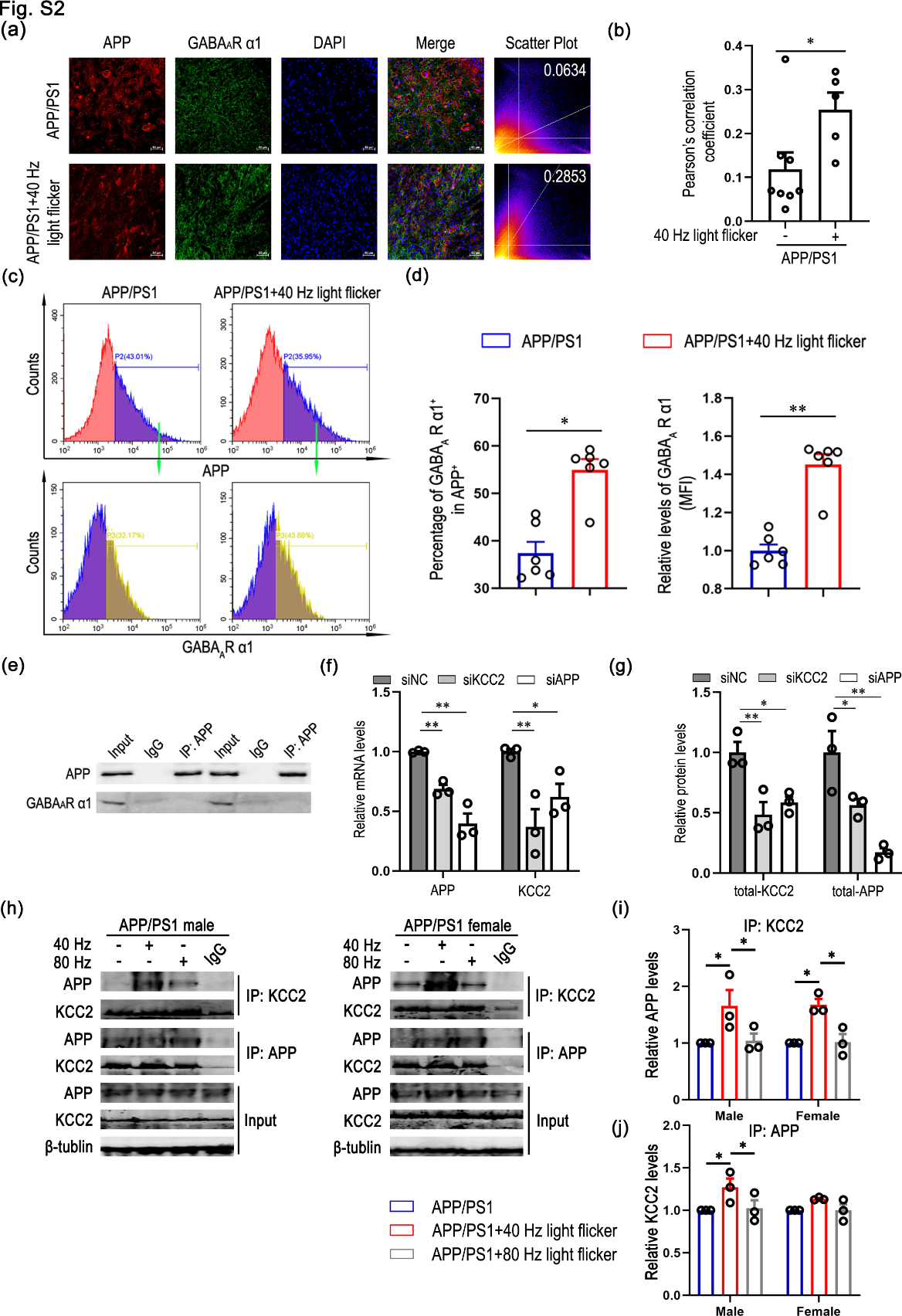
**

**Figure S2:** Enhancement of APP trafficking to plasma membrane by gamma frequency light flicker maintains total and surface KCC2 levels, thereby stabilizing GABA_A_R α1 subunit on the plasma membrane. (a) Immunohistochemistry with anti-APP (red) and GABA_A_R α1 (green) in cerebral cortex of 6-month-old APP/PS1 after 7 days of 1 h/day dark or 40 Hz light flicker, scale bar, 50 μm. (b) Pearson's correlation coefficient analysis of APP and GABA_A_R α1 (*n* = 8 mice in APP/PS1 group, *n* = 5 mice in APP/PS1 with 40 Hz flicker group). Data are presented as mean ± SEM. **p* < 0.05 vs. indicated group, by unpaired *t* test. (c-d) Gates P2 (blue gate) and P3 (khaki gate) for surface APP and GABA_A_R α1 were determined respectively in the unstained group, and the number of APP^+^ cells (gate P2) was allowed to count 10000 statistically in each experimental group, and the percentage number of GABA_A_R α1^+^ cells and relative mean fluorescence intensity (MFI) levels of surface GABA_A_R α1 in the gate P2 (APP^+^ cells) were analyzed on a CytoFLEX flow cytometer, using CytExpert software (*n* = 6 mice/group). Data are presented as mean ± SEM. **p* < 0.05 vs. indicated group, ***p* < 0.01 vs. indicated group, by unpaired *t* test. (e) GABA_A_R α1 subunit does not coimmunoprecipitate with APP. (f) APP and KCC2 mRNA levels were detected by quantitative real-time PCR after treatment with KCC2 siRNA, or APP siRNA (at least three independent experiments). The primer of KCC2: 5'-GCCATTCACAGCTTCCCCAAACTTG-3' (reverse), 5'-CCACCTCTGCTGTCTACATCAGCTC-3' (forward); the primer of APP: 5'-GCCAAGACATCGTCGGAGTAGT-3' (reverse), 5'-TCCGTGTGATCTACGAGCGCAT-3' (forward); the primer of GAPDH: 5'-CCTTGACTGTGCCGTTGAATTT-3' (reverse), 5'-GCAAAGTGGAGATTGTTGCCAT-3' (forward). Data are presented as mean ± SEM. **p* < 0.05 vs. indicated group, ***p* < 0.01 vs. indicated group, by two-way ANOVA with *Tukey's post hoc* multiple comparisons test. (g) Quantification of total-KCC2, total-APP levels in siNC, siKCC2, and siAPP treatment group (at least three independent experiments). Data are presented as mean ± SEM. **p* < 0.05 vs. indicated group, ***p* < 0.01 vs. indicated group, by two-way ANOVA with *Tukey's post hoc* multiple comparisons test. (h) Representative western blots showing co-immunoprecipitation with both KCC2 and APP antibodies in cerebral cortex of 6-month-old male and female APP/PS1 mice following dark, 40 Hz, or 80 Hz flicker stimulation (*n* = 3 mice per group). Data are presented as mean ± SEM. **p* < 0.05 vs. indicated group, by two-way ANOVA with *Tukey's post hoc* multiple comparisons test. (i) Relative immunoreactivity of APP normalized to KCC2 (IP: KCC2). (j) Relative immunoreactivity of KCC2 normalized to APP (IP: APP).


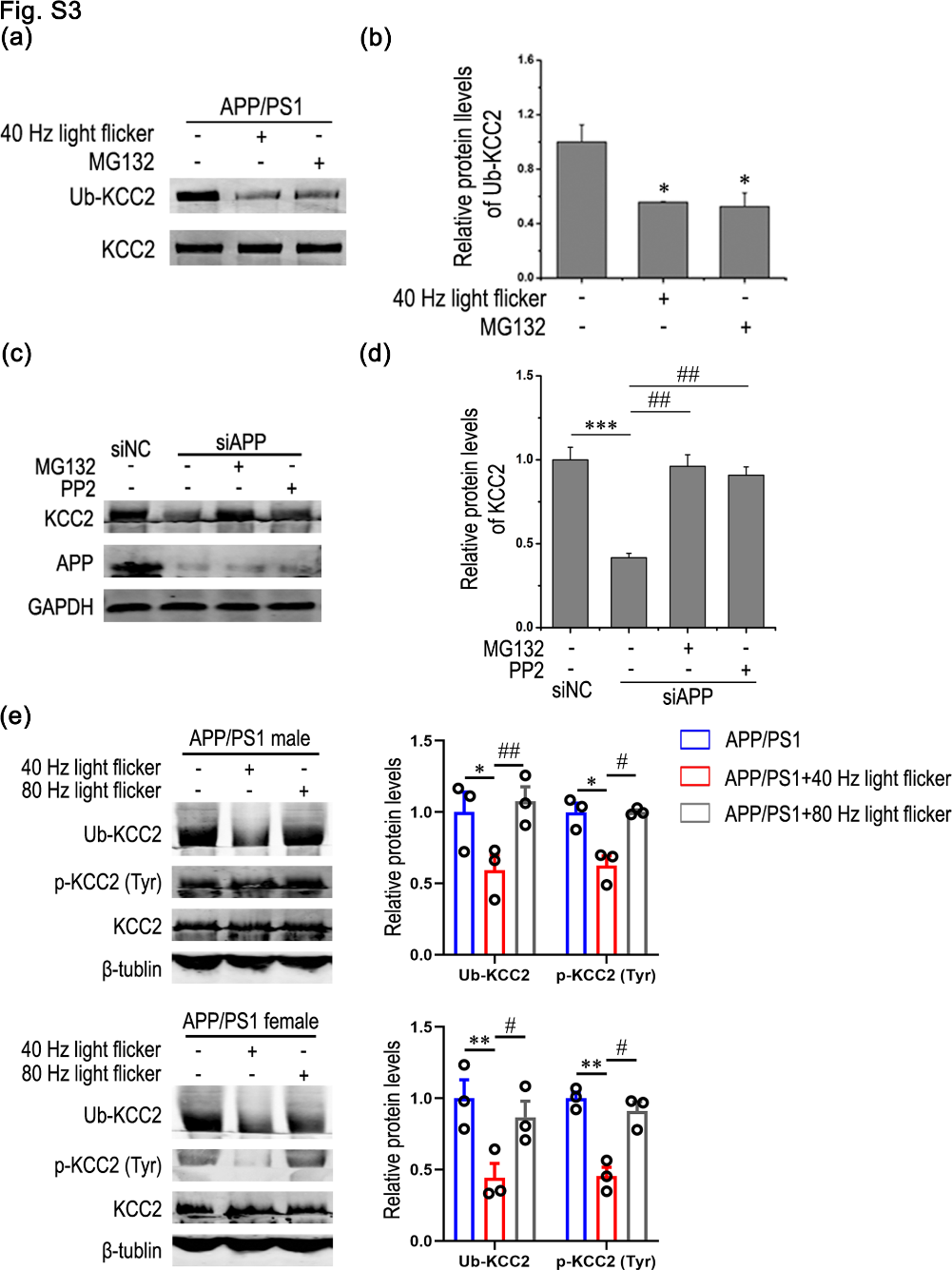


**Figure S3:** Gamma frequency light flicker inhibits tyrosine phosphorylation and degradation of KCC2 through APP. (a) Representative western blots of ubiquitinated KCC2 (Ub-KCC2) in 6-month-old APP/PS1 mice under the treatment of 7 days of 1 h/day 40 Hz light flicker or MG132. (b) Relative immunoreactivity of Ub-KCC2 normalized to KCC2 (*n* = 3 to 4 mice per group). All the data are reported as mean ± SEM. **p* < 0.05 vs. control group, by two-way ANOVA with *Tukey's post hoc* multiple comparisons test. (c-d) Western blot analysis of KCC2 and APP expressions after treatment with MG132 or PP2 in APP deficient group (silencing with APP siRNA). All the data are reported as mean ± SEM of four independent experiments. ****p* < 0.001 vs. the control group, ##*p* < 0.01 vs. indicated group, by one-way ANOVA with *Tukey's post hoc* multiple comparisons test. (e) Representative western blots of ubiquitinated KCC2 (Ub-KCC2) and phosphorylated KCC2 on tyrosine (p-KCC2 (Tyr)) in 6-month-old male and female APP/PS1 mice following dark, 40 Hz, or 80 Hz flicker stimulation (*n* = 3 mice per group). All the data are reported as mean ± SEM. **p* < 0.05 vs. APP/PS1 group, ***p* < 0.01 vs. APP/PS1 group, #*p* < 0.05 vs. indicated group, ##*p* < 0.01 vs. indicated group, by two-way ANOVA with *Tukey's post hoc* multiple comparisons test.


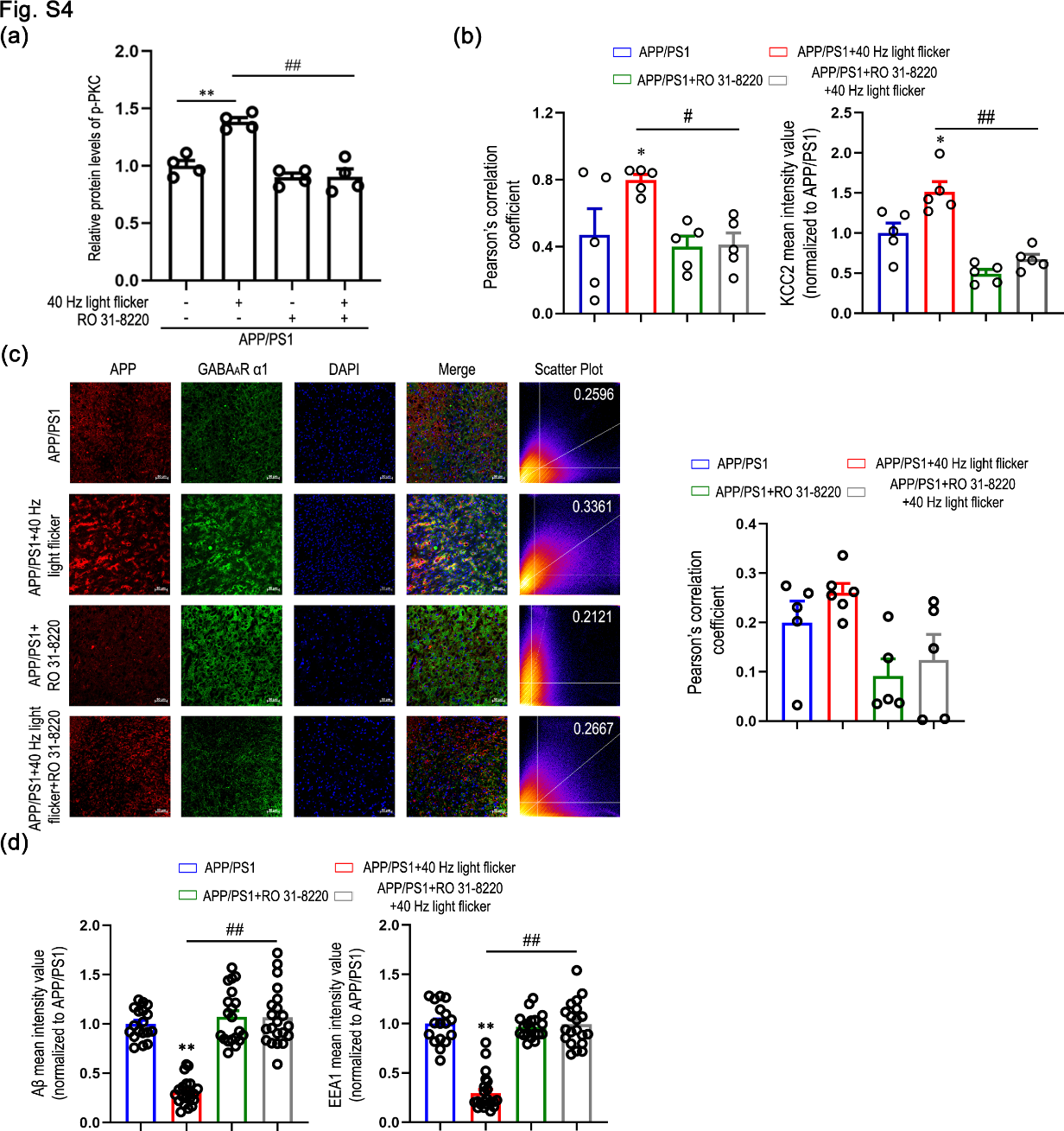


**Figure S4:** Gamma frequency light flicker promotes the interaction between APP and KCC2 by activating the PKC signaling pathway, thus stabilizing both on the cell surface. (a) Quantification of p-PKC levels in cerebral cortex of 6-month-old APP/PS1 mice exposed to 7 days of dark, 40 Hz light flicker, RO 31-8220 (6 mg/kg/d, s.c), RO 31-8220 (6 mg/kg/d, s.c) with 40 Hz flicker (*n* = 4 mice per group). Data are presented as mean ± SEM. **p* < 0.05 vs. APP/PS1 group; ***p* < 0.01 vs. APP/PS1 group; *#p* < 0.05 vs. indicated group; *##p* < 0.01 vs. indicated group, by two-way ANOVA with *Tukey's post hoc* multiple comparisons test. (b) Pearson's correlation coefficient analysis of APP and KCC2, and quantification of KCC2 levels in cortex of 6-month-old APP/PS1 treated with dark, 40 Hz light flicker, RO 31-8220 (6 mg/kg/d, s.c), RO 31-8220 (6 mg/kg/d, s.c) with 40 Hz flicker for 7 days (*n* = 5 mice per group). Data are presented as mean ± SEM. **p* < 0.05 vs. APP/PS1 group; *#p* < 0.05 vs. indicated group; *##p* < 0.01 vs. indicated group, by two-way ANOVA with *Tukey's* *post hoc* multiple comparisons test. (c) Immunohistochemistry with anti-APP (red) and GABA_A_R α1 (green) in cerebral cortex of 6-month-old APP/PS1 after 7 days of 1 h/day dark, 40 Hz flicker, RO 31-8220 (6 mg/kg/d, s.c), RO 31-8220 (6 mg/kg/d, s.c) with 40 Hz flicker. Pearson's correlation coefficient analysis of APP and GABA_A_R α1 in cortex of 6-month-old APP/PS1 treated with 7 days of dark, 40 Hz light flicker, RO 31-8220 (6 mg/kg/d, s.c), RO 31-8220 (6 mg/kg/d, s.c) with 40 Hz flicker (*n* = 5-6 mice per group). (d) Relative immunoreactivity of Aβ and EEA1 in cortex normalized to APP/PS1 controls (*n* = 18-21 slices from 6 to 7 mice per group). Data are presented as mean ± SEM. **p* < 0.05 vs. APP/PS1 group; ***p* < 0.01 vs. APP/PS1 group; *#p* < 0.05 vs. indicated group; *##p* < 0.01 vs. indicated group, by two-way ANOVA with *Tukey's post hoc* multiple comparisons test.

**
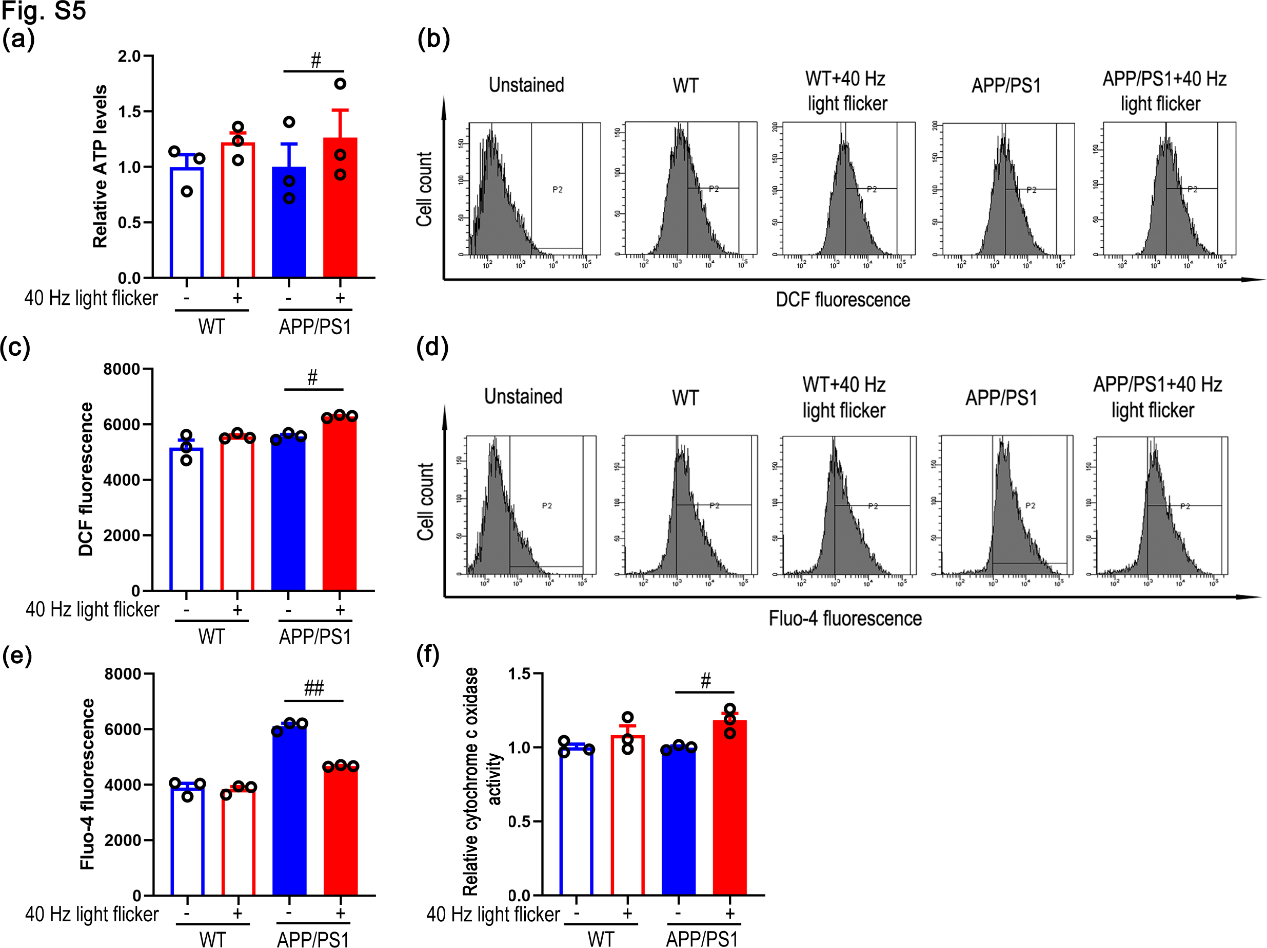
**

**Figure S5:** Gamma frequency light flicker activates PKC by increasing the production of ATP. (a) Quantification of ATP levels in 6-month-old WT or APP/PS1 mice treated with or without 7 days of 1 h/day 40 Hz light flicker (*n* = 3 mice per group). Flow cytometry **b** and quantification **c** of ROS production in 6-month-old WT or APP/PS1 mice treated with or without 7 days of 1 h/day 40 Hz light flicker (*n* = 3 mice per group). Flow cytometry **d** and quantification **e** of Ca^2+^ production in 6-month-old WT or APP/PS1 mice after 7 days of 1 h/day dark or 40 Hz light flicker (*n* = 3 mice per group). (f) Quantification of cytochrome c oxidase activity in 6-month-old WT or APP/PS1 mice treated with or without 7 days of 1 h/day 40 Hz light flicker (*n* = 3 mice per group). Data are presented as mean ± SEM. *#p* < 0.05 vs. indicated group; *##p* < 0.01 vs. indicated group, by one-way ANOVA with *Tukey's post hoc*.

**
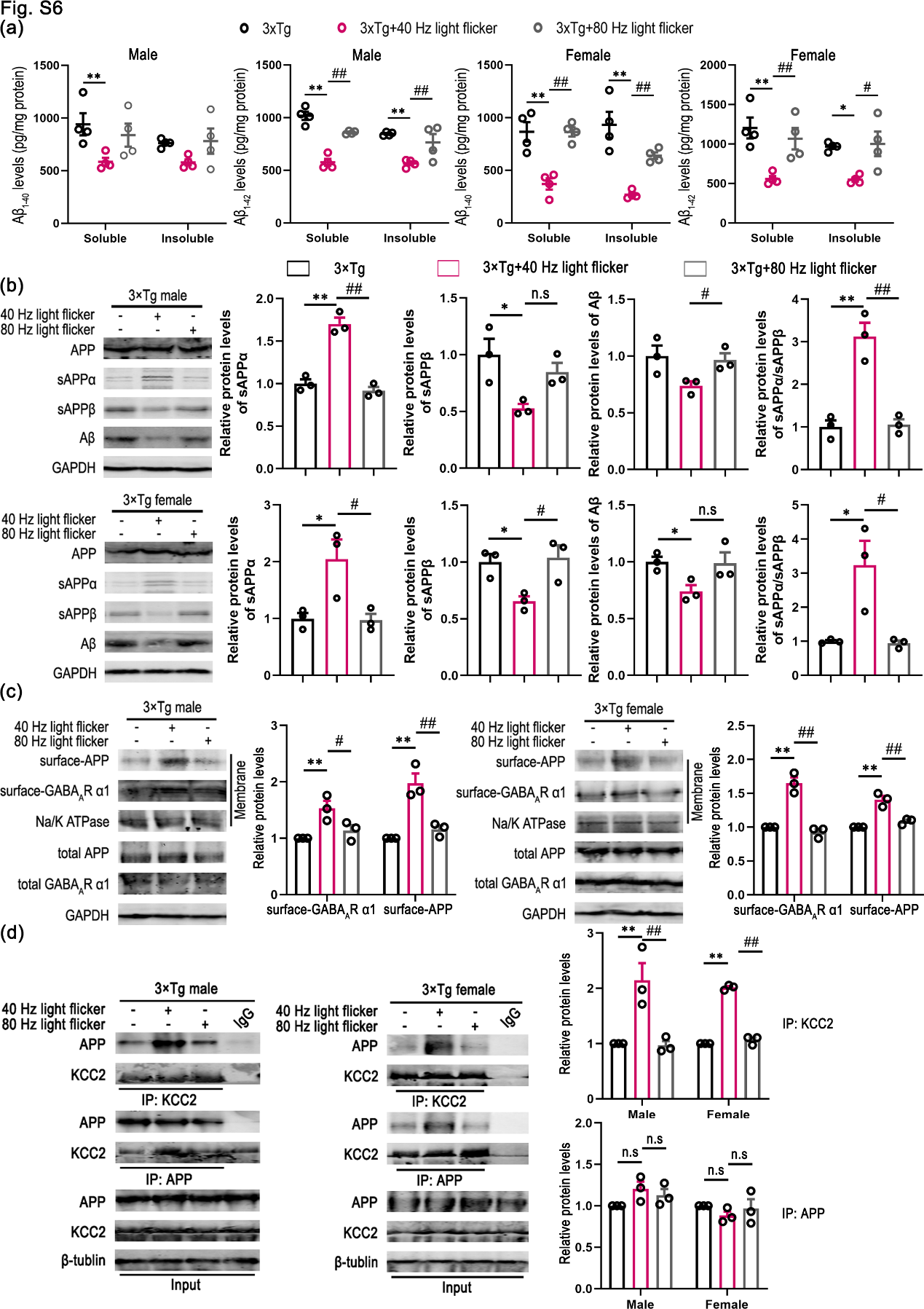
**

**Figure S6:** Gamma frequency light flicker increased anchoring of APP to the plasma-membrane to reduce amyloid load in 3×Tg mice. (a) Soluble and insoluble Aβ_1-40_ and Aβ_1-42_ levels in cortex of 10-month-old male and female 3×Tg mice after 7 days of 1 h per day dark, 40 Hz or 80 Hz flicker were performed by ELISA, respectively (*n* = 4 mice per group). Data are presented as mean ± SEM. **p* < 0.05 vs. 3×Tg group, ***p* < 0.01 vs. 3×Tg group, *#p* < 0.05 vs. indicated group, *##p* < 0.01 vs. indicated group, by two-way ANOVA with *Tukey's* *post hoc* multiple comparisons test. (b) Representative western blot showing levels of APP, sAPPα, sAPPβ and Aβ in cerebral cortex of male and female 3×Tg mice with 7 days of 1 h/day dark, 40 Hz, or 80 Hz light flicker (*n* = 3 mice per group). And quantification of relative sAPPα/sAPPβ ratios in 3×Tg male and female group following dark, 40 Hz, or 80 Hz flicker stimulation, respectively. Data are presented as mean ± SEM. **p* < 0.05 vs. 3×Tg group, ***p* < 0.01 vs. 3×Tg group, *#p* < 0.05 vs. indicated group, *##p* < 0.01 vs. indicated group, *n.s* = not significant, by two-way ANOVA with *Tukey's post hoc* multiple comparisons test. (c) Representative immunoblots and quantification of surface-APP and surface-GABA_A_R α1 levels in 3×Tg male and female group following dark, 40 Hz, or 80 Hz flicker stimulation, respectively (*n* = 3 mice per group). Data are presented as mean ± SEM. ***p* < 0.01 vs. 3×Tg group, *#p* < 0.05 vs. indicated group, *##p* < 0.01 vs. indicated group, by two-way ANOVA with *Tukey's post hoc* multiple comparisons test. (d) Representative western blots showing co-immunoprecipitation with both KCC2 and APP antibodies in cortex of male and female 3×Tg mice following dark, 40 Hz, or 80 Hz flicker stimulation (*n* = 3 mice per group). Data are presented as mean ± SEM. ***p* < 0.01 vs. 3×Tg group, *##p* < 0.01 vs. indicated group, *n.s* = not significant, by two-way ANOVA with *Tukey's post hoc* multiple comparisons test.


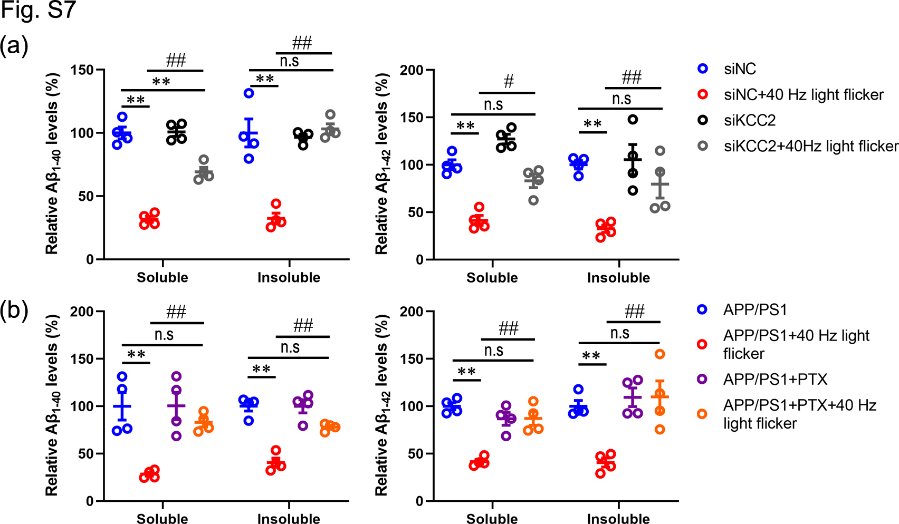


**Figure S7:** KCC2 and GABA_A_ receptors play an important role in the reduction of amyloid load by 40 Hz light flicker. (a) Relative soluble and insoluble Aβ_1-40_ and Aβ_1-42_ levels in APP/PS1 treated with siNC, siNC+40 Hz light flicker, siKCC2, or siKCC2+40 Hz light flicker were performed by ELISA (*n* = 4). Data are presented as mean ± SEM. ***p* < 0.01 vs. siNC group, *#p* < 0.05 vs. indicated group, *##p* < 0.01 vs. indicated group, *n.s* = not significant, by two-way ANOVA with *Tukey's post hoc* multiple comparisons test. (b) Relative soluble and insoluble Aβ_1-40_ and Aβ_1-42_ levels in APP/PS1, APP/PS1+40 Hz light flicker, APP/PS1+PTX, and APP/PS1+PTX+40 Hz light flicker were performed by ELISA (*n* = 4). Data are presented as mean ± SEM. ***p* < 0.01 vs. APP/PS1 group, *##p* < 0.01 vs. indicated group, *n.s* = not significant, by two-way ANOVA with *Tukey's post hoc* multiple comparisons test. PTX: picrotoxin, 0.18 mg/kg.


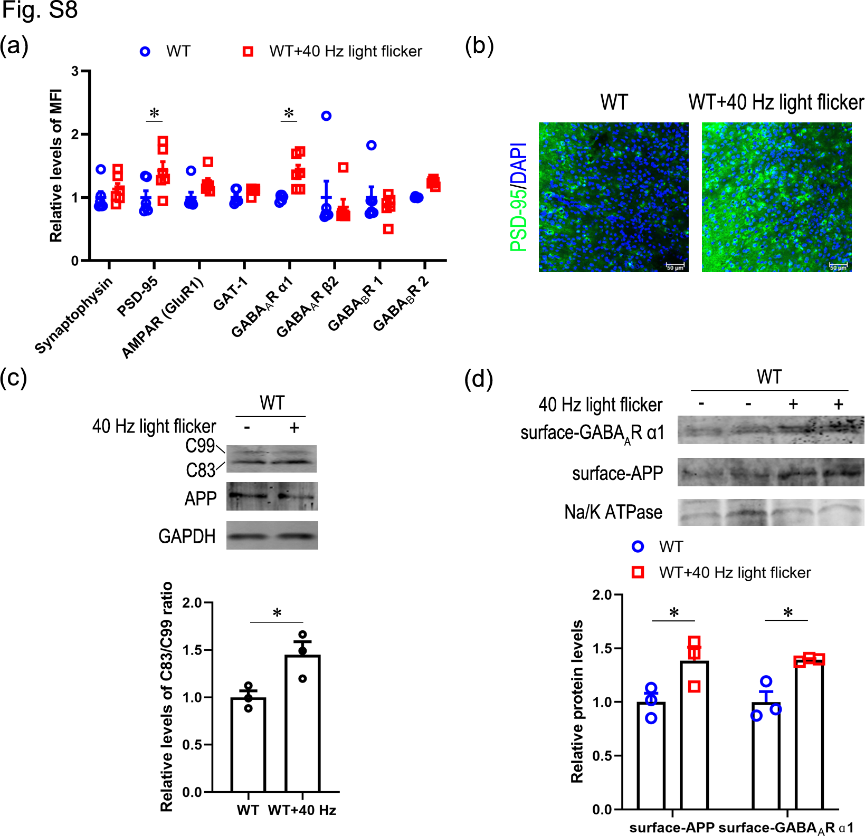


**Figure S8:** The effect of gamma frequency stimulation on WT mice. (a) The fluorescence levels of synaptophysin, PSD-95, AMPAR (GluR1), GAT-1, GABA_A_R α1, GABA_A_R β2, GABA_B_R1, GABA_B_R2 was detected by flow cytometry in different groups (*n* = 6 mice per group). MFI: mean fluorescence intensity. Data are presented as mean ± SEM. **p* < 0.05 vs. WT group, by Student's *t* test. (b) Representative images of PSD-95 (green) and DAPI labeling (blue) of cell nuclei in WT group after 7 days of 1 h/day dark, or 40 Hz light flicker, scale bar: 50 μm. (c) Representative western blot showing levels of APP, and APP-CTF in WT group with 7 days of 1 h/day dark, or 40 Hz light flicker (*n* = 3 mice per group). And quantification of relative C83/C99 ratio in WT group following dark, or 40 Hz stimulation. Data are presented as mean ± SEM. **p* < 0.05 vs. WT group, by Student's *t* test. (d) Representative immunoblots and quantification of surface-APP and surface-GABA_A_R α1 levels in WT group following dark, or 40 Hz stimulation (*n* = 3 mice per group). Data are presented as mean ± SEM. **p* < 0.05 vs. WT group, by Student's *t* test.


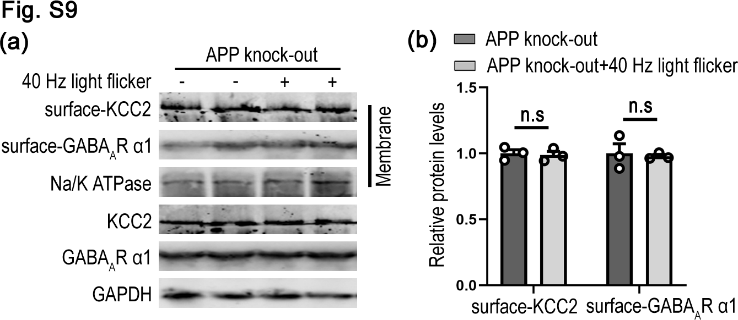


**Figure S9:** The effects of gamma frequency light flicker on surface KCC2 and GABA_A_R α1 levels in APP knock-out. (a) Representative immunoblots of surface KCC2 and GABA_A_R α1 levels in APP knock-out under dark, or 40 Hz light flicker treatment. (b) Quantification of surface-KCC2 levels and surface-GABA_A_R α1 levels (*n* = 3). Data are presented as mean ± SEM. *n.s* = not significant, by Student's *t* test.
